# Evaluation of Effectiveness of Autocidal Gravid Ovitraps for Preventing Zika Virus Infection, Puerto Rico, USA

**DOI:** 10.3201/eid3204.251206

**Published:** 2026-04

**Authors:** Zachary J. Madewell, Sandra J. Kiplagat, India Kellum, Matthew J. Lozier, Olga Lorenzi, Janice Perez-Padilla, Freddy A. Medina, Jorge L. Muñoz-Jordán, Laura E. Adams, Gabriela Paz-Bailey, Stephen H. Waterman, Roberto Barrera, Tyler M. Sharp

**Affiliations:** Centers for Disease Control and Prevention, San Juan, Puerto Rico, USA (Z.J. Madewell, S.J. Kiplagat, I. Kellum, M.J. Lozier, O. Lorenzi, J. Perez-Padilla, F.A. Medina, J.-L. Muñoz-Jordán, L.E. Adams, G. Paz-Bailey, S.H. Waterman, R. Barrera, T.M. Sharp); US Public Health Service Commissioned Corps, Rockville, Maryland, USA (L.E. Adams, T.M. Sharp)

**Keywords:** Zika virus, vector-borne infections, viruses, Aedes aegypti, mosquitoes, AGO mass trapping, vector control, Zika seroprevalence, Caribbean, Puerto Rico, United States

## Abstract

*Aedes aegypti* mosquitoes drive arboviral outbreaks in tropical regions. Zika virus (ZIKV), linked to congenital and neurologic complications, caused a major outbreak in Puerto Rico, USA, in 2016, infecting ≈26% of the population. Autocidal gravid ovitraps (AGOs), pesticide-free devices targeting gravid *Ae. aegypti* mosquitoes, have been shown to reduce transmission of another arbovirus, chikungunya. During March–May 2017, we conducted a household-based serosurvey in 4 demographically similar communities in southeastern Puerto Rico, 2 with long-term AGO deployment (≈85% coverage) and 2 without, to assess effects of AGOs on ZIKV transmission. Among 271 participants >5 years of age, ZIKV seroprevalence was much lower in intervention than nonintervention communities (9.6% vs. 20.0%). Protective effects were strongest among older adults, larger households (>4 persons), and persons spending more time at home. Although study design and measurement limitations could limit generalizability of results, our findings support AGOs as sustainable nonchemical tools for reducing ZIKV infections.

Throughout the Americas, dengue virus (DENV), chikungunya virus (CHIKV), and Zika virus (ZIKV), transmitted by *Aedes aegypti* mosquitoes, cause periodic outbreaks (*1*,*2*). Those arboviruses often cocirculate, overwhelming health systems in tropical regions (*1*–*3*). During a 2015–2016 epidemic, ZIKV gained global attention for its links to congenital Zika syndrome, microcephaly, and Guillain-Barré syndrome (*4*). In Puerto Rico, USA, >71,000 suspected cases and >39,000 laboratory-confirmed ZIKV infections were reported during that period (*5*,*6*).

Conventional *Aedes* spp. mosquito control strategies, such as insecticide spraying, habitat removal, and community education, face growing limitations. Insecticide resistance is widespread, spraying is costly and labor-intensive, and sustained community engagement for source reduction is often difficult to maintain (*7*,*8*). Those challenges have spurred interest in alternative tools, such as the autocidal gravid ovitrap (AGO), a pesticide-free device developed by the Centers for Disease Control and Prevention (CDC) to attract and trap gravid female *Ae. aegypti* mosquitoes (*9*). Once inside the AGO, mosquitoes are unable to escape, reducing breeding populations without chemical insecticides. AGOs require infrequent maintenance and have sustained effects when deployed at scale (*8*,*10*).

Long-term community AGO deployment has been shown to reduce *Ae. aegypti* mosquito densities by up to 80% (*11*–*13*). During the 2014–2015 chikungunya outbreak in Puerto Rico, communities using AGOs had lower mosquito densities and 5-fold lower CHIKV infection rates, suggesting that AGOs can meaningfully disrupt arbovirus transmission (*14*). Although CHIKV and ZIKV share a vector, differences in epidemic timing, asymptomatic infection rates, and behavioral responses might influence intervention effectiveness. AGO effectiveness for reducing ZIKV infection risk has not been well studied. Given the possible severe outcomes from ZIKV infection, evaluating the protective effect of AGOs is critical for informing public health strategies.

Although no confirmed ZIKV infections have been reported in Puerto Rico since 2019, competent mosquito vectors and risk for reintroduction persist. In 2024, Puerto Rico experienced its first major dengue outbreak in >10 years, in which >6,000 confirmed cases and 11 deaths occurred (*15*–*17*). That outbreak underscores the ongoing threat of mosquitoborne viruses, their substantial economic burden, and the need for sustainable control strategies (*18*,*19*). We evaluated the effectiveness of AGOs in reducing ZIKV infection and *Ae. aegypti* mosquito abundance in Puerto Rico by comparing ZIKV seroprevalence and mosquito abundance between communities with and without AGOs. 

## Methods

### Study Setting

We conducted this study in 4 communities already participating in a long-term entomologic trial in the Salinas and Guayama municipalities on Puerto Rico’s southeastern coast. In 2015, the population of Salinas was 30,114 and the population of Guayama was 43,700; both had population densities of 434–672 persons per square mile (*13*). Both municipalities have young (median age ≈36 years) populations, ≈15% of whom are >65 years of age (*13*). The municipalities also have near equal sex distribution, and >50% of households are below the poverty line (*13*).

Community-level data showed that the 4 study sites were small, semiurban neighborhoods with comparable population sizes, household densities, and occupancy rates (Appendix Table 1). Average household size was 2.6–3.6 persons. Most homes were single-story with patios or gardens, and architecture and climate were similar across sites. All communities had piped water and waste removal services, including sewer or septic coverage depending on location (*9*,*13*,*20*). Those indicators support baseline demographic and infrastructural comparability of intervention and nonintervention sites.

The 2 intervention communities (La Margarita, Villodas) are geographically buffered by vegetation or roads (200–500 meters), reducing mosquito movement from adjacent areas (*20*). The nonintervention communities (Arboleda, La Playa) without AGOs are embedded in larger urban zones. Although not randomized, we selected sites that were demographically and environmentally comparable on the basis of census and field data (*13*,*21*). All 4 sites have had continuous mosquito surveillance since 2012. Prior analyses confirmed that differences in *Ae. aegypti* mosquito abundance between intervention and nonintervention areas emerged only after trap deployment (*14*,*22*). Consistent with prior evaluations, we observed similar female *Ae. aegypti* mosquito abundance during the predeployment baseline period, October–December 2011, in the original paired communities of La Margarita and Villodas (Appendix Figure 1). A CHIKV serosurvey in those sites found no systematic demographic or household-level differences (*13*). Other community-based serosurveys in Puerto Rico have shown consistent ZIKV, DENV, and CHIKV seroprevalence patterns, and variation was driven more by age and household factors than geography (*23*,*24*). Together, those findings confirmed sustained reductions in *Ae. aegypti* mosquitoes in intervention areas and limited underlying differences, supporting the inference that observed ZIKV effects were unlikely to reflect underlying community characteristics (*13*,*20*,*25*).

### AGO Intervention

AGOs attract and capture gravid female *Ae. aegypti* mosquitoes (*9*,*10*,*20*). Each household in intervention communities received 3 traps, maintained every 2 months by trained staff. Deployment began during 2011–2013 and ultimately covered ≈85% of households. Previous studies documented that intervention communities experienced marked and sustained reductions in *Ae. aegypti* mosquito abundance compared with nonintervention sites (*21*). Weekly entomologic surveillance using sentinel AGOs provided data on *Ae. aegypti* mosquito abundance, which we linked to participant infection status (*9*,*20*,*26*) (Appendix). 

### Study Design and Sampling

We conducted a cross-sectional, community-based serosurvey during March–May 2017 to assess ZIKV infection among residents in the 4 communities. We conducted the serosurvey 6–9 months after peak ZIKV transmission in Puerto Rico during August 2016 to capture infections from the outbreak period. The survey overlapped with the seasonal arbovirus activity trough (March–April), when incident infections are uncommon (*17*). Therefore, IgM detection in this survey reflects infections acquired during the 2016 epidemic rather than new infections occurring at the time of sampling. Although the serosurvey was conducted in 2017, analysis and reporting were delayed because of competing public health response priorities and the time required for data harmonization, quality assurance, and linkage to longitudinal entomologic surveillance.

For sampling, we used a stratified random design, assigning a unique identifier to each residential structure and randomly selecting households to achieve ≈28.5% coverage of the total population. We chose 28.5% coverage to balance statistical power with operational feasibility for household-based venous blood collection. The 28%–30% coverage target was successfully applied in prior arboviral serosurveys in the same study communities and yielded representative samples for demographic and household characteristics (*13*,*25*). Field teams visited each selected household up to 3 times to recruit participants. If a household was vacant or the head-of-household remained unavailable after 3 visits, we randomly replaced that household to meet enrollment targets.

Eligible participants included all residents >5 years of age who slept in the selected household for >4 of the previous 7 nights. We excluded children <5 years of age because of the difficulty of venous blood collection and ethical considerations of venipuncture in that age group. Although younger children can provide valuable information on recent arbovirus circulation in endemic settings, ZIKV was newly introduced in Puerto Rico in 2015–2016; thus, all age groups were susceptible, and infection risk was broadly distributed. Our primary aim was to assess community-level ZIKV infection prevalence across the general population after the epidemic, which we could accomplish by including participants >5 years of age. Participant selection was independent of household AGO presence; in intervention communities, households were neither included nor excluded based on whether AGOs were installed at that specific residence. 

We obtained written informed consent from all adult participants. Persons 15–20 years of age provided written assent with parental or guardian permission, and children 5–14 years of age provided verbal assent with written parental or guardian permission. All participants provided blood specimens, regardless of reported symptoms, and completed structured questionnaires on demographic and housing characteristics, mosquito prevention practices, and recent illness history (Appendix). This study was reviewed and approved by the CDC Institutional Review Board (protocol no. 6800).

### Laboratory Testing

Field teams collected venous blood specimens and transported specimens on the same day to the CDC Dengue Branch (Division of Vector-Borne Diseases, National Center for Emerging and Zoonotic Infectious Diseases) in San Juan, Puerto Rico. Upon arrival, we centrifuged samples to separate serum, then aliquoted and stored serum at –20°C until testing.

We tested serum specimens for ZIKV IgM by using the CDC Zika IgM antibody capture ELISA (Zika MAC-ELISA), following CDC instructions, and tested for DENV IgM by using the DENV Detect IgM capture ELISA (InBios International, Inc., https://inbios.com), following manufacturer instructions. The Zika MAC-ELISA has high sensitivity and specificity for recent ZIKV infection in dengue-endemic settings, although some cross-reactivity with other flaviviruses, particularly DENV, can occur (*27*,*28*). To minimize misclassification, our primary outcome defined ZIKV infection as ZIKV IgM–positive and DENV IgM–negative results, an approach supported by evaluations of the Zika MAC-ELISA showing that IgM reactivity is strongest for the homotypic virus in dengue-endemic settings (*27*). We also conducted a sensitivity analysis by including participants testing positive for ZIKV IgM, DENV IgM, or both. We did not test for DENV or ZIKV IgG because available assays show substantial cross-reactivity among ZIKV-exposed persons in dengue-endemic settings, limiting the usefulness of those assays for distinguishing prior ZIKV from prior DENV infection (*29*). 

### Statistical Analysis

We estimated prevalence ratios (PRs) for ZIKV infection by using Poisson regression with robust SEs, adjusting for age, sex, and time spent at home. We explored effect modification across demographic and household subgroups. Sensitivity analyses included broader arbovirus IgM outcomes and models incorporating entomologic data. We conducted all analyses in R version 4.4.2 (The R Project for Statistical Computing, https://www.r-project.org) (Appendix). 

## Results

### Study Population

A total of 330 participants from 242 households completed the serosurvey: 71 households from Arboleda (nonintervention), 79 from La Margarita (intervention), 40 from La Playa (nonintervention), and 52 from Villodas (intervention). We selected households from among 1,228 total residential structures, of which 1,014 (82.6%) were occupied during enumeration (Appendix Figure 2). We prioritized household sampling by structures that participated in a prior 2015–2016 CHIKV serosurvey, then randomly selected replacement households to meet enrollment targets.

Of the 330 enrolled participants, we excluded 55 because of indeterminate serology results: 32 hemolyzed samples, 17 equivocal results, and 6 nonspecific results. We excluded another 4 participants who tested DENV IgM–positive. Thus, we included a total of 271 (82.1%) participants from 208 households: 65 households in Arboleda, 65 in La Margarita, 33 in La Playa, and 45 in Villodas. Among included participants, 136 (50.2%) lived in intervention communities and 135 (49.8%) lived in nonintervention communities. Participants’ median age was 59 (IQR 46–69) years; 165 (60.9%) were female and 106 (39.1%) were male (Table 1; Appendix Table 2). Most had lived in their communities for >10 years, and household characteristics were similar between groups.

**Table 1:** Characteristics of participants in an evaluation of effectiveness of autocidal gravid ovitraps for preventing Zika virus infection, Puerto Rico, USA*

Characteristics	Total, n = 271	Participant data		p value†
		Intervention, n = 136	Nonintervention, n = 135	
Age group, y, n = 267				0.283
<20	18 (6.7)	9 (6.7)	9 (6.8)	
20–39	31 (11.6)	19 (14.2)	12 (9.0)	
40–59	86 (32.2)	47 (35.1)	39 (29.3)	
>60	132 (49.4)	59 (44.0)	73 (54.9)	
Median age, y (IQR)	59 (46–69)	59 (43–69)	60 (49–69)	0.324
Sex				0.242
F	165 (60.9)	88 (64.7)	77 (57.0)	
M	106 (39.1)	48 (35.3)	58 (43.0)	
Years in community, n = 269				0.244
0–19	104 (38.7)	56 (41.2)	48 (36.1)	
20–39	116 (43.1)	52 (38.2)	64 (48.1)	
>40	49 (18.2)	28 (20.6)	21 (15.8)	
Median years (IQR) in community, n = 269	24 (12–35)	21 (13–35)	29 (12–34)	0.307
Acute illness with rash, fever, or joint pain since November 2015, n = 270				0.271
Y	58 (21.5)	25 (18.4)	33 (24.6)	
N	212 (78.5)	111 (81.6)	101 (75.4)	
Household characteristics				
No. persons in household				0.516
1	60 (22.1)	30 (22.1)	30 (22.2)	
2	101 (37.3)	47 (34.6)	54 (40.0)	
3	52 (19.2)	25 (18.4)	27 (20.0)	
>4	58 (21.4)	34 (25.0)	24 (17.8)	
Median no. (IQR) persons in household	2 (2–3)	2 (2–3)	2 (2–3)	0.454
No. children <5 years of age				0.081
0	250 (92.3)	121 (89.0)	129 (95.6)	
1	17 (6.3)	13 (9.6)	4 (3.0)	
2	4 (1.5)	2 (1.5)	2 (1.5)	
Annual household income, USD, n = 238				0.059
<25,000	173 (72.7)	88 (73.3)	85 (72.0)	
26,000–50,000	46 (19.3)	18 (15.0)	28 (23.7)	
51,000–75,000	16 (6.7)	11 (9.2)	5 (4.2)	
>76,000	3 (1.3)	3 (2.5)	0 (0.0)	
Dwelling type				0.393
One-story house	248 (91.5)	122 (89.7)	126 (93.3)	
Two-story house	23 (8.5)	14 (10.3)	9 (6.7)	
No. screened windows/doors, n = 270				0.789
1	139 (51.5)	70 (51.9)	69 (51.1)	
2	87 (32.2)	45 (33.3)	42 (31.1)	
3	44 (16.3)	20 (14.8)	24 (17.8)	
Air conditioning use				0.312
In all rooms	42 (15.5)	18 (13.2)	24 (17.8)	
Only in bedroom at night	140 (51.7)	68 (50.0)	72 (53.3)	
None	89 (32.8)	50 (36.8)	39 (28.9)	
Frequency of open windows/doors				0.496
Always	96 (35.4)	46 (33.8)	50 (37.0)	
Only during the day	116 (42.8)	63 (46.3)	53 (39.3)	
Only at night	7 (2.6)	2 (1.5)	5 (3.7)	
Never	52 (19.2)	25 (18.4)	27 (20.0)	
Mosquito exposure				
Weekly hours at home				0.827
<24	19 (7.0)	9 (6.6)	10 (7.4)	
25–60	91 (33.6)	48 (35.3)	43 (31.9)	
61–84	161 (59.4)	79 (58.1)	82 (60.7)	
Median weekly hours (IQR) at home	73 (42–84)	72 (42–84)	74 (44–84)	0.536

*Values are no. (%) except as indicated. Intervention communities had autocidal gravid ovitraps installed; nonintervention communities did not. IQR, interquartile range. †χ^2^ test was used for comparison of percentages, Fisher exact test was for comparison of categories with >1 value <5, and Mann-Whitney U test was used for medians.

The secondary sensitivity analysis included an expanded sample of 297 participants with valid ZIKV or DENV IgM results, including those who were DENV IgM–positive. That population had similar demographic and household characteristics to the primary analytic sample (Appendix Table 3).

### Baseline Characteristics

Characteristics among participants in intervention and nonintervention communities did not differ substantially, including for age, sex, number of household residents, and duration of residence (Table 1; Appendix Table 2). However, participants in nonintervention communities reported higher levels of mosquito exposure than participants in intervention communities, including more frequent daily bites (24.6% vs. 8.1%; p = 0.004) and being bitten at home (79.3% vs. 66.9%; p = 0.031). Citronella use was more common in nonintervention areas (31.9% vs. 16.2%; p = 0.004), suggesting greater perceived or actual mosquito abundance or differing perceptions of citronella’s effectiveness relative to other control methods. Other prevention behaviors, such as repellent or coil use, were similar across groups. We observed similar patterns in the sensitivity analysis (Appendix Table 3).

### ZIKV Seroprevalence

**Figure 1:**
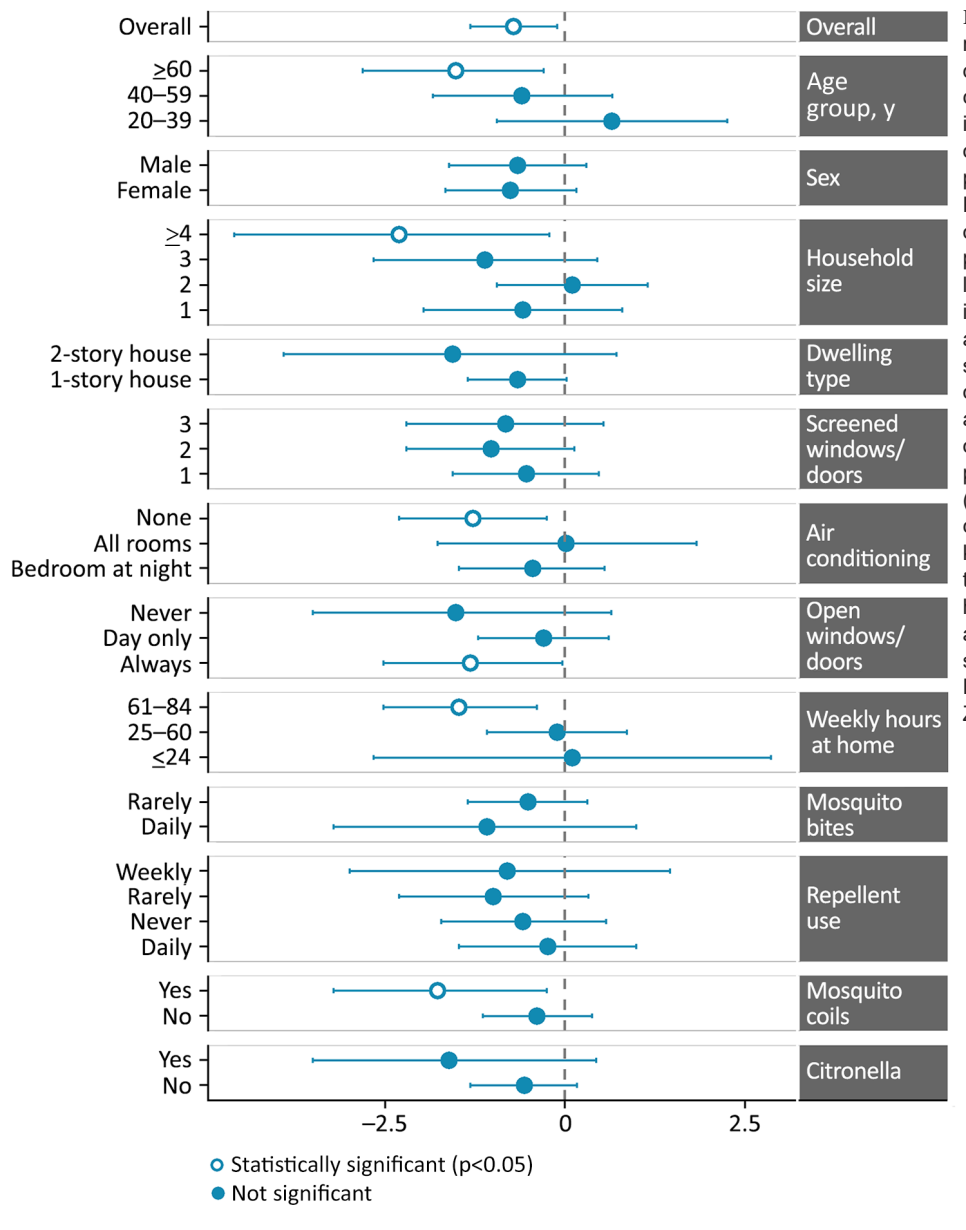
Adjusted prevalence ratios for ZIKV seropositivity by demographic and behavioral characteristic among participants in an evaluation of effectiveness of autocidal gravid ovitraps for preventing Zika virus infection, Puerto Rico, USA. Values left of 0 (corresponding to adjusted prevalence ratio <1) indicate lower ZIKV seroprevalence in intervention communities. log-adjusted prevalence ratios show statistically significant (p<0.05) overall seroprevalence reduction, and specific reductions among older adults (>60 years of age), participants in larger households (>4 persons), those without air conditioning, those who always kept windows or doors open, and those spending >61 hours/week at home. Use of mosquito coils was also associated with reduced ZIKV seropositivity in intervention areas. Error bars represent 95% CIs. ZIKV, Zika virus.

Overall, we detected recent ZIKV infection in 40 of the 271 participants, corresponding to 14.8% seroprevalence. Seroprevalence was much lower (9.6%, 13/136) in intervention communities than nonintervention communities (20.0%, 27/135). Overall crude PR was 0.48 (95% CI 0.26–0.89); after adjusting for confounders, the adjusted PR (aPR) was 0.49 (95% CI 0.27–0.90) (Figure 1; Appendix Table 4).

Lower ZIKV seroprevalence in intervention communities was consistent across most demographic and behavioral subgroups (Figure 1; Appendix Table 4). Among participants >60 years of age, seroprevalence was 5.1% in intervention and 23.3% in nonintervention communities (aPR 0.22 [95% CI 0.06–0.75]). We observed similar protective associations among participants in larger households (>4 residents; aPR 0.10 [95% CI 0.01–0.81]), without air conditioning (aPR 0.28 [95% CI 0.10–0.78]), and those who used mosquito coils (aPR 0.17 [95% CI 0.04–0.78]), although subgroup sizes were small.

Among participants who always kept windows or doors open, those in intervention communities had lower seroprevalence than those in nonintervention communities (6.5% vs. 24.0%; aPR 0.27 [95% CI 0.08–0.97]). Similarly, those spending >61 hours/week at home in intervention areas had lower seroprevalence compared with those in nonintervention areas (5.1% vs. 22.0%). In intervention communities, predicted infection probability rose with time at home, peaking at 52 hours per week before declining (Appendix Figure 3). In contrast, infection probability in nonintervention communities increased steadily, peaking at 84 hours.

Among the 297 participants in the expanded sensitivity analysis, 41 (13.8%) tested ZIKV IgM–positive and 5 (1.7%) tested DENV IgM–positive (Appendix Tables 3, 4). Overall arbovirus seroprevalence remained much lower in intervention communities than in nonintervention communities (9.2% vs. 21.5%; aPR 0.44 [95% CI 0.24–0.78]) (Appendix Table 5). The strongest protective associations persisted among older adults (aPR 0.21 [95% CI 0.07–0.62]), participants without air conditioning (aPR 0.25 [95% CI 0.09–0.69]), and those in larger households (aPR 0.10 [95% CI 0.01–0.80]), supporting the robustness of the primary findings.

### Perceptions of AGO Effectiveness

Among participants in intervention communities, 105/136 (77.2%) reported a reduction in household mosquito density related to AGOs. Few reported an increase (3.7%, n = 5) or no change (8.1%, n = 11) in mosquito density, and 11.0% (n = 15) were unsure or did not respond to that question.

### ZIKV Seropositivity and Acute Febrile Illness

Among ZIKV-seropositive participants, 37.5% (15/40) reported experiencing an acute febrile illness since November 2015, compared with 18.7% (43/231) of ZIKV-seronegative participants (p = 0.014) (Table 2). Seropositive participants more frequently reported common Zika symptoms than seronegative participants, including rash (27.5% vs. 8.7%), fever (30.0% vs. 15.2%), and joint pain (32.5% vs. 16.5%) (p≤0.040). However, care-seeking (20.0% seropositive vs. 12.1% seronegative; p = 0.270) and hospitalization (2.5% seropositive vs. 0.4% seronegative; p = 0.682) were infrequent and did not differ significantly by serostatus.

**Table 2:** Reported acute febrile illness and symptom profiles among participants in an evaluation of effectiveness of autocidal gravid ovitraps for preventing Zika virus infection, Puerto Rico, USA *

Characteristics	Overall, n = 270†	ZIKV-positive, n = 40	ZIKV-negative, n = 230	p value‡
Acute febrile illness	58 (21.5)	15 (37.5)	43 (18.7)	0.014
Rash	31 (11.5)	11 (27.5)	20 (8.7)	0.002
Fever	47 (17.4)	12 (30.0)	35 (15.2)	0.040
Joint pain	51 (18.9)	13 (32.5)	38 (16.5)	0.030
Sought medical care for illness	36 (13.3)	8 (20.0)	28 (12.2)	0.275
Hospitalized for illness	2 (0.7)	1 (2.5)	1 (0.4)	0.684

*Values are no. (%). ZIKV, Zika virus. †One ZIKV-negative participant with missing information on acute febrile illness and related symptom history was excluded from this table. ‡χ^2^ test was used for comparison of percentages and Fisher exact test was for comparison of categories with >1 value <5.

### Entomologic Trends and ZIKV Seropositivity Association 

**Figure 2:**
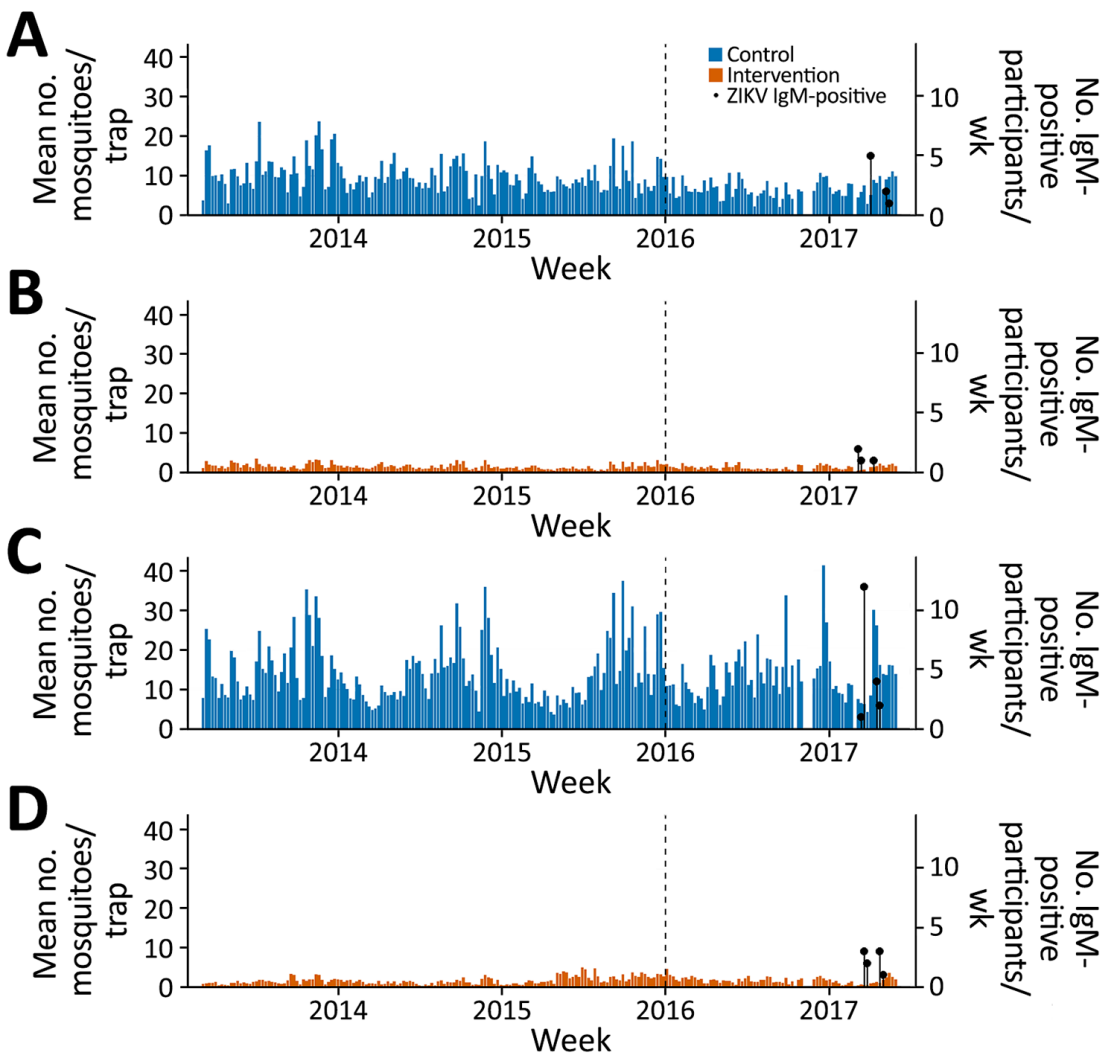
Weekly *Aedes aegypti* mosquito abundance from an evaluation of effectiveness of autocidal gravid ovitraps (AGOs) for preventing Zika virus infection, Puerto Rico, USA. Mean mosquito counts per surveillance trap are shown weekly for each of the 4 study communities: A) Arboleda; B) La Margarita; C) La Playa; D) Villodas. Intervention communities had AGOs. Black lollipop markers indicate the weekly number of ZIKV IgM–positive participants identified during the 2017 postepidemic serosurvey. Dashed vertical lines indicate first known ZIKV case in Puerto Rico on December 31, 2015. Mosquito abundance remained substantially lower in intervention communities throughout the epidemic period, aligning with the reduced ZIKV seroprevalence observed in those areas. Scales for the y-axes differ substantially to underscore patterns but do not permit direct comparisons. ZIKV, Zika virus.

During the ZIKV epidemic, January 2016–May 2017, mean weekly *Ae. aegypti* mosquito abundance per surveillance trap was substantially lower in intervention than nonintervention communities (1.40 [95% CI 1.28–1.52] vs. 9.98 [95% CI 8.98–11.00] mosquitoes per trap) (Figure 2). In Poisson regression models adjusted for age category, sex, and hours spent at home, higher mosquito abundance was positively associated with ZIKV seropositivity. Each additional female mosquito captured per trap-week was associated with a 4% increase in ZIKV seropositivity risk at a 2-week lag (risk ratio [RR] 1.044 [95% CI 1.011–1.077]; p = 0.008) (Table 3). Associations at shorter lag times were similar in magnitude and reached statistical significance at a 1-week lag (RR 1.028 [95% CI 1.003–1.053]; p = 0.026), but not at 0 lag. Formal interaction tests provided no evidence that associations differed by intervention status (Appendix Table 6).

**Table 3:** Association between lagged *Aedes aegypti* female mosquito abundance and ZIKV IgM seropositivity in an evaluation of effectiveness of autocidal gravid ovitraps for preventing Zika virus infection, Puerto Rico, USA *

Lag, wks	Adjusted RR (95% CI)†	p value	p for interaction‡
0	1.028 (0.996–1.062)	0.091	0.115
1	1.028 (1.003–1.053)	0.026	0.776
2	1.044 (1.011–1.077)	0.008	0.400

*Reflects test for effect modification by community intervention status. RR, risk ratio; ZIKV, Zika virus. †RRs were estimated by using Poisson regression with a log link and robust (sandwich) SEs. Lagged mosquito abundance was modeled as a continuous predictor (per +1 female per trap-week). Lags 0–2 represent mosquito abundance in the week of specimen collection (lag 0) and 1–2 weeks prior (lags 1–2). Adjusted models include age category, sex, and hours spent at home (community) categories. ‡Interaction p values are Wald tests from pooled models including a mosquito abundance × intervention status interaction term using robust standard errors.

## Discussion

In this community-based serosurvey, AGO deployment was associated with lower ZIKV seroprevalence and higher mosquito suppression in intervention communities compared with nonintervention communities. Residents in intervention communities had approximately half the ZIKV seroprevalence of residents in nonintervention communities. However, because we did not conduct a randomized evaluation and seroprevalence was measured 6–9 months after peak transmission, residual confounding and differential misclassification might have contributed to the observed difference. Associations appeared stronger in some subgroups (e.g., older adults, larger households, and participants spending more time at home), although subgroup estimates were imprecise and should be interpreted cautiously. Overall, these findings are consistent with, but do not establish, a protective association between AGOs and lower peridomestic vector exposure and arboviral infection risk (*30*).

Our findings build on research demonstrating sustained *Ae. aegypti* mosquito population reductions in the same communities where AGOs have been maintained for nearly a decade (*8*). Similar effects were observed in northern Mexico and North Carolina, where mass trapping reduced *Aedes* spp. mosquito abundance and shifted mosquito populations toward younger, less infectious females (*31*,*32*). During Puerto Rico’s 2014–2015 chikungunya outbreak, CHIKV seroprevalence in AGO intervention areas was half that of nonintervention areas (*13*,*25*). This study extends that evidence to ZIKV, revealing a positive association between reduced mosquito abundance and seropositivity, particularly at a 2-week lag, consistent with the ZIKV incubation period (*33*). Our results also align with entomologic surveillance, which showed frequent ZIKV detection in *Ae. aegypti* mosquito pools from untreated sites but rarely in AGO communities during the 2016 epidemic (*14*). Even modest increases in vector density could elevate short-term infection risk, aligning with findings suggesting DENV transmission is unlikely when weekly female *Ae. aegypti* mosquito densities remain <4/trap (*30*). Stronger apparent protection among older persons and those spending more time at home is consistent with the peridomestic biting behavior of *Ae. aegypti* mosquitoes (*34*,*35*) and a household-level mechanism of protection (*18*). The lack of protection among younger adults might reflect increased mobility and mosquito exposure outside the home, which is concerning for pregnant women, who face increased risk for ZIKV complications. However, few participants in our study were pregnant, limiting our ability to directly assess those differences. If AGOs provide less protection for more mobile persons, complementary strategies, including personal protection, prenatal counseling, and risk messaging, might be needed during outbreaks.

Our results highlight the potential of nonchemical vector control tools to reduce arbovirus transmission. Other interventions have demonstrated reductions in mosquito densities, but few have shown population-level impacts on human infection (*36*,*37*). AGOs offer a pesticide-free, community-accepted alternative that requires infrequent maintenance and is well suited to semiurban settings where indoor mosquito biting is common and insecticide resistance limits traditional approaches (*18*). Compared with aerial spraying or *Wolbachia*-based bacterial releases, AGOs are less resource-intensive, but large-scale deployment would require sustained funding, logistical coordination, and public-sector capacity. The observed association with lower ZIKV infection supports continued evaluation of AGOs as part of integrated vector management.

The relationship between time spent at home and ZIKV risk differed by community type, and infection probability rose more steeply in nonintervention areas. That finding aligns with evidence that human mobility influences arboviral exposure and should be considered in intervention evaluations (*38*–*40*). We also observed protective associations among participants without air conditioning and those who kept windows or doors open, suggesting AGOs could be particularly beneficial in households with higher mosquito exposure. Mosquito coil use appeared beneficial in intervention communities, highlighting potential added value in combining AGOs with personal protection tools in integrated strategies.

The first limitation of this study is that sampling occurred 6–9 months after peak ZIKV transmission; thus, waning IgM might have underestimated cumulative incidence. However, ZIKV IgM can persist for >12–25 months; one study reported detectable IgM in >70% of ZIKV-infected persons at 12–19 months (*41*), suggesting that our survey likely captured most infections from the 2016 outbreak. Nonetheless, if infection timing differed systematically between community types, differential IgM detectability could have biased between-community comparisons (e.g., earlier infections in intervention communities could accentuate differences due to waning seroprevalence, whereas earlier infections in nonintervention communities would tend to attenuate differences). Second, all participants were sampled during the same period using the same protocol, but we cannot exclude temporal differences in infection timing as a contributor to observed differences in IgM seroprevalence. Third, we did not measure IgG, which would have provided information on baseline flavivirus seroprevalence. However, DENV transmission was minimal during the study period, as documented by passive and enhanced surveillance that reported no laboratory-confirmed dengue cases in 2017 (*3*,*16*), reducing the likelihood that cocirculating dengue or DENV-ZIKV cross-reactivity materially biased IgM results. In dengue-endemic settings, conventional DENV-like particle IgG assays show substantial cross-reactivity among ZIKV-exposed persons, limiting their specificity for distinguishing prior DENV versus ZIKV infection. In Puerto Rico, DENV and ZIKV are the only flaviviruses with sustained human transmission, and no ZIKV circulation was documented before the 2015–2016 epidemic. We selected demographically and environmentally comparable intervention and nonintervention communities, and longstanding entomologic surveillance and prior household-based serosurveys of CHIKV and DENV did not indicate large systematic differences between communities. Those data provide some reassurance that major imbalances in underlying immunity are not obvious; however, we cannot rule out meaningful community-level differences in baseline exposure risk or other unmeasured factors that might influence infection risk. Any residual flavivirus cross-immunity would be expected to be similar across communities and would tend to bias estimates toward the null, consistent with cohort data from Nicaragua showing that prior DENV infection reduced symptomatic ZIKV disease but did not alter overall ZIKV infection risk (symptomatic and inapparent combined) (*42*). Fourth, several self-reported indicators related to mosquito exposure differed by community type, but because we collected data on those indicators after AGOs were implemented for several years and after the epidemic, they cannot be interpreted as definitive baseline differences and might reflect intervention-related changes in exposure or reporting. As a nonrandomized community comparison, residual confounding from unmeasured differences between communities (e.g., fine-scale environmental conditions, housing characteristics, human mobility patterns, or uptake of personal protective behaviors) could partially or fully explain the observed seroprevalence differences. Finally, the modest sample size limited precision, particularly for subgroup estimates, so evidence of effect modification should be cautiously interpreted.

Despite those limitations, use of a validated ELISA, exclusion of DENV IgM–positive participants in the primary analysis, minimal DENV transmission during the study period, and consistent sensitivity analyses increase confidence that the observed association is not solely attributable to assay limitations. Most ZIKV-seropositive participants did not report illness or care-seeking, underscoring the value of serologic surveillance in capturing asymptomatic or unrecognized infections. 

In conclusion, by linking entomologic control with human health outcomes, this study contributes to the evidence for sustained *Aedes* spp. mosquito vector control to reduce ZIKV transmission. Amid increasing arbovirus outbreaks and rising concerns about insecticide resistance, integrating AGOs into broader vector control programs could help close the gap between entomologic impact and human health benefit.

Appendix Additional information on an evaluation of effectiveness of autocidal gravid ovitraps for preventing Zika virus infection, Puerto Rico.

## References

[1] Paixão ES, Teixeira MG, Rodrigues LC. Zika, chikungunya and dengue: the causes and threats of new and re-emerging arboviral diseases. BMJ Glob Health. 2018;3:e000530. 10.1136/bmjgh-2017-000530 29435366 PMC5759716

[2] Patterson J, Sammon M, Garg M. Dengue, Zika and chikungunya: emerging arboviruses in the New World. West J Emerg Med. 2016;17:671–9. 10.5811/westjem.2016.9.30904 27833670 PMC5102589

[3] Madewell ZJ, Hernandez-Romieu AC, Wong JM, Zambrano LD, Volkman HR, Perez-Padilla J, et al. Sentinel enhanced dengue surveillance system—Puerto Rico, 2012–2022. MMWR Surveill Summ. 2024;73:1–29. 10.15585/mmwr.ss7303a1 38805389 PMC11152364

[4] Musso D, Gubler DJ. Zika virus. Clin Microbiol Rev. 2016;29:487–524. 10.1128/CMR.00072-15 27029595 PMC4861986

[5] Sharp TM, Quandelacy TM, Adams LE, Aponte JT, Lozier MJ, Ryff K, et al. Epidemiologic and spatiotemporal trends of Zika virus disease during the 2016 epidemic in Puerto Rico. PLoS Negl Trop Dis. 2020;14:e0008532. 10.1371/journal.pntd.0008532 32956416 PMC7529257

[6] Quandelacy TM, Healy JM, Greening B, Rodriguez DM, Chung KW, Kuehnert MJ, et al. Estimating incidence of infection from diverse data sources: Zika virus in Puerto Rico, 2016. PLOS Comput Biol. 2021;17:e1008812. 10.1371/journal.pcbi.1008812 33784311 PMC8034731

[7] Dusfour I, Vontas J, David JP, Weetman D, Fonseca DM, Corbel V, et al. Management of insecticide resistance in the major *Aedes* vectors of arboviruses: advances and challenges. PLoS Negl Trop Dis. 2019;13:e0007615. 10.1371/journal.pntd.0007615 31600206 PMC6786541

[8] Barrera R, Harris A, Hemme RR, Felix G, Nazario N, Muñoz-Jordan JL, et al. Citywide control of *Aedes aegypti* (Diptera: Culicidae) during the 2016 Zika epidemic by integrating community awareness, education, source reduction, larvicides, and mass mosquito trapping. J Med Entomol. 2019;56:1033–46. 10.1093/jme/tjz009 30753539 PMC6597296

[9] Barrera R, Amador M, Acevedo V, Caban B, Felix G, Mackay AJ. Use of the CDC autocidal gravid ovitrap to control and prevent outbreaks of *Aedes aegypti* (Diptera: Culicidae). J Med Entomol. 2014;51:145–54. 10.1603/ME13096 24605464 PMC4631065

[10] Mackay AJ, Amador M, Barrera R. An improved autocidal gravid ovitrap for the control and surveillance of *Aedes aegypti.* Parasit Vectors. 2013;6:225. 10.1186/1756-3305-6-225 23919568 PMC3750875

[11] Obregón JA, Ximenez MA, Villalobos EE, de Valdez MRW. Vector mosquito surveillance using Centers for Disease Control and Prevention autocidal gravid ovitraps in San Antonio, Texas. J Am Mosq Control Assoc. 2019;35:178–85. 10.2987/18-6809.1 31647715

[12] Montenegro D, Martinez L, Tay K, Hernandez T, Noriega D, Barbosa L, et al. Usefulness of autocidal gravid ovitraps for the surveillance and control of *Aedes* (*Stegomyia*) *aegypti* (Diptera: Culicidae) in eastern Colombia. Med Vet Entomol. 2020;34:379–84. 10.1111/mve.12443 32232987

[13] Sharp TM, Lorenzi O, Torres-Velásquez B, Acevedo V, Pérez-Padilla J, Rivera A, et al. Autocidal gravid ovitraps protect humans from chikungunya virus infection by reducing *Aedes aegypti* mosquito populations. PLoS Negl Trop Dis. 2019;13:e0007538. 10.1371/journal.pntd.0007538 31344040 PMC6657827

[14] Barrera R, Amador M, Acevedo V, Beltran M, Muñoz JL. A comparison of mosquito densities, weather and infection rates of *Aedes aegypti* during the first epidemics of chikungunya (2014) and Zika (2016) in areas with and without vector control in Puerto Rico. Med Vet Entomol. 2019;33:68–77. 10.1111/mve.12338 30225842 PMC6378603

[15] Ware-Gilmore F, Rodriguez DM, Ryff MPHK, Torres JM, Velez MP, Torres-Toro CT, et al. Dengue outbreak and response—Puerto Rico, 2024. MMWR Morb Mortal Wkly Rep. 2025;74:54–60. 10.15585/mmwr.mm7405a1 39977371 PMC12370255

[16] Rodriguez DM, Madewell ZJ, Torres JM, Rivera A, Wong JM, Santiago GA, et al. Epidemiology of dengue—Puerto Rico, 2010–2024. MMWR Morb Mortal Wkly Rep. 2024;73:1112–7. 10.15585/mmwr.mm7349a1 39666586 PMC11637419

[17] Thayer MB, Marzan-Rodriguez M, Torres Aponte J, Rivera A, Rodriguez DM, Madewell ZJ, et al. Dengue epidemic alert thresholds for surveillance and decision-making in Puerto Rico: development and prospective application of an early warning system using routine surveillance data. BMJ Open. 2025;15:e106182. 10.1136/bmjopen-2025-106182 40998432 PMC12481368

[18] Barrera R. New tools for *Aedes* control: mass trapping. Curr Opin Insect Sci. 2022;52:100942. 10.1016/j.cois.2022.100942 35667560 PMC9413017

[19] Camprubí-Ferrer D, Thayer MB, Madewell ZJ, Mac McCullough J, Sánchez-González L, Rivera A, et al. Economic burden of dengue in Puerto Rico, 2010–2023. Infect Dis Poverty. 2026;15:15. 10.1186/s40249-026-01412-1 41588438 PMC12837138

[20] Barrera R, Amador M, Acevedo V, Hemme RR, Félix G. Sustained, area-wide control of *Aedes aegypti* using CDC autocidal gravid ovitraps. Am J Trop Med Hyg. 2014;91:1269–76. 10.4269/ajtmh.14-0426 25223937 PMC4257658

[21] Hemme RR, Smith EA, Felix G, White BJ, Diaz-Garcia MI, Rodriguez D, et al. Multi-year mass-trapping with autocidal gravid ovitraps has limited influence on insecticide susceptibility in *Aedes aegypti* (Diptera: Culicidae) from Puerto Rico. J Med Entomol. 2022;59:314–9. 10.1093/jme/tjab162 34536077

[22] Barrera R, Felix G, Acevedo V, Amador M, Rodriguez D, Rivera L, et al. Impacts of hurricanes Irma and Maria on *Aedes aegypti* populations, aquatic habitats, and mosquito infections with dengue, chikungunya, and Zika viruses in Puerto Rico. Am J Trop Med Hyg. 2019;100:1413–20. 10.4269/ajtmh.19-0015 30963992 PMC6553919

[23] Adams LE, Sánchez-González L, Rodriguez DM, Ryff K, Major C, Lorenzi O, et al. Risk factors for infection with chikungunya and Zika viruses in southern Puerto Rico: a community-based cross-sectional seroprevalence survey. PLoS Negl Trop Dis. 2022;16:e0010416. 10.1371/journal.pntd.0010416 35696355 PMC9191703

[24] Adams LE, Hitchings MDT, Medina FA, Rodriguez DM, Sánchez-González L, Moore H, et al. Previous dengue infection among children in Puerto Rico and implications for dengue vaccine implementation. Am J Trop Med Hyg. 2023;109:413–9. 10.4269/ajtmh.23-0091 37308104 PMC10397428

[25] Lorenzi OD, Major C, Acevedo V, Perez-Padilla J, Rivera A, Biggerstaff BJ, et al. Reduced incidence of chikungunya virus infection in communities with ongoing *Aedes aegypti* mosquito trap intervention studies—Salinas and Guayama, Puerto Rico, November 2015–February 2016. MMWR Morb Mortal Wkly Rep. 2016;65:479–80. 10.15585/mmwr.mm6518e3 27171600

[26] Madewell ZJ, Hemme RR, Adams L, Barrera R, Waterman SH, Johansson MA. Comparing vector and human surveillance strategies to detect arbovirus transmission: a simulation study for Zika virus detection in Puerto Rico. PLoS Negl Trop Dis. 2019;13:e0007988. 10.1371/journal.pntd.0007988 31877132 PMC6948821

[27] Medina FA, Vila F, Premkumar L, Lorenzi O, Paz-Bailey G, Alvarado LI, et al. Capacity of a multiplex IgM antibody capture ELISA to differentiate Zika and dengue virus infections in areas of concurrent endemic transmission. Am J Trop Med Hyg. 2022;106:585–92. 10.4269/ajtmh.20-1651 34929668 PMC8832915

[28] Machado Portilho M, de Moraes L, Kikuti M, Jacob Nascimento LC, Galvão Reis M, Sampaio Boaventura V, et al. Accuracy of the Zika IgM antibody capture enzyme-linked immunosorbent assay from the Centers for Disease Control and Prevention (CDC Zika MAC-ELISA) for diagnosis of Zika virus infection. Diagnostics (Basel). 2020;10:835. 10.3390/diagnostics10100835 33080935 PMC7603149

[29] Gaspar-Castillo C, Rodríguez MH, Ortiz-Navarrete V, Alpuche-Aranda CM, Martinez-Barnetche J. Structural and immunological basis of cross-reactivity between dengue and Zika infections: implications in serosurveillance in endemic regions. Front Microbiol. 2023;14:1107496. 10.3389/fmicb.2023.1107496 37007463 PMC10063793

[30] Barrera R, Acevedo-Soto V, Ruiz-Valcarcel J, Medina J, Rivera R, Otero L, et al. Defining *Aedes aegypti* density thresholds for preventing human arboviral infections. Acta Trop. 2025;267:107688. 10.1016/j.actatropica.2025.107688 40480602 PMC13207729

[31] Aguilar-Durán JA, Hamer GL, Reyes-Villanueva F, Fernández-Santos NA, Uriegas-Camargo S, Rodríguez-Martínez LM, et al. Effectiveness of mass trapping interventions using autocidal gravid ovitraps (AGO) for the control of the dengue vector, *Aedes* (*Stegomyia*) *aegypti*, in Northern Mexico. Parasit Vectors. 2024;17:344. 10.1186/s13071-024-06361-y 39154005 PMC11330617

[32] Figurskey AC, Hollingsworth B, Doyle MS, Reiskind MH. Effectiveness of autocidal gravid trapping and chemical control in altering abundance and age structure of *Aedes albopictus.* Pest Manag Sci. 2022;78:2931–9. 10.1002/ps.6917 35417621 PMC9321977

[33] Krow-Lucal ER, Biggerstaff BJ, Staples JE. Estimated incubation period for Zika virus disease. Emerg Infect Dis. 2017;23:841–5. 10.3201/eid2305.161715 28277198 PMC5403043

[34] Facchinelli L, Badolo A, McCall PJ. Biology and behaviour of *Aedes aegypti* in the human environment: opportunities for vector control of arbovirus transmission. Viruses. 2023;15:636. 10.3390/v15030636 36992346 PMC10053764

[35] Madewell ZJ, Sosa S, Brouwer KC, Juárez JG, Romero C, Lenhart A, et al. Associations between household environmental factors and immature mosquito abundance in Quetzaltenango, Guatemala. BMC Public Health. 2019;19:1729. 10.1186/s12889-019-8102-5 31870343 PMC6929347

[36] Achee NL, Grieco JP, Vatandoost H, Seixas G, Pinto J, Ching-Ng L, et al. Alternative strategies for mosquito-borne arbovirus control. PLoS Negl Trop Dis. 2019;13:e0006822. 10.1371/journal.pntd.0006822 30605475 PMC6317787

[37] Wilson AL, Courtenay O, Kelly-Hope LA, Scott TW, Takken W, Torr SJ, et al. The importance of vector control for the control and elimination of vector-borne diseases. PLoS Negl Trop Dis. 2020;14:e0007831. 10.1371/journal.pntd.0007831 31945061 PMC6964823

[38] Stoddard ST, Forshey BM, Morrison AC, Paz-Soldan VA, Vazquez-Prokopec GM, Astete H, et al. House-to-house human movement drives dengue virus transmission. Proc Natl Acad Sci U S A. 2013;110:994–9. 10.1073/pnas.1213349110 23277539 PMC3549073

[39] Wesolowski A, Qureshi T, Boni MF, Sundsøy PR, Johansson MA, Rasheed SB, et al. Impact of human mobility on the emergence of dengue epidemics in Pakistan. Proc Natl Acad Sci U S A. 2015;112:11887–92. 10.1073/pnas.1504964112 26351662 PMC4586847

[40] Phillips MT, Sánchez-González L, Shragai T, Rodriguez DM, Major CG, Johansson MA, et al. Quantifying the relationship between arboviral infection prevalence and human mobility patterns among participants of the Communities Organized to Prevent Arboviruses cohort (COPA) in southern Puerto Rico. PLoS Negl Trop Dis. 2023;17:e0011840. 10.1371/journal.pntd.0011840 38100525 PMC10756524

[41] Griffin I, Martin SW, Fischer M, Chambers TV, Kosoy O, Falise A, et al. Zika virus IgM detection and neutralizing antibody profiles 12–19 months after illness onset. Emerg Infect Dis. 2019;25:299–303. 10.3201/eid2502.181286 30666931 PMC6346474

[42] Gordon A, Gresh L, Ojeda S, Katzelnick LC, Sanchez N, Mercado JC, et al. Prior dengue virus infection and risk of Zika: a pediatric cohort in Nicaragua. PLoS Med. 2019;16:e1002726. 10.1371/journal.pmed.1002726 30668565 PMC6342296

