# Supplementary material for: Evaluation of Effectiveness of Autocidal Gravid Ovitraps for Preventing Zika Virus Infection, Puerto Rico, USA
Source: Emerg Infect Dis. 2026 Apr;32(4):510–20. doi: 10.3201/eid3204.251206 (PMC13094846; doi:10.3201/eid3204.251206)
Supplement: Appendix — Additional information on an evaluation of effectiveness of autocidal gravid ovitraps for preventing Zika virus infection, Puerto Rico. [file 25-1206-Techapp-s1.pdf]

Article DOI: https://doi.org/10.3201/eid3204.251206

*EID cannot ensure accessibility for supplementary materials supplied by authors. Readers who have difficulty accessing supplementary content should contact the authors for assistance.*

# Evaluation of Effectiveness of Autocidal Gravid Ovitraps for Preventing Zika Virus Infection, Puerto Rico, USA

## Appendix

## Supplemental Methods

### Autocidal Gravid Ovitrap Intervention

AGOs are pesticide-free devices developed to reduce *Ae. aegypti* populations by targeting gravid females, which transmit arboviruses (*1*–*3*). Each trap comprises a 19-L black polyethylene pail with ~10 L of water and a 30-g hay packet to create a fermented infusion that attracts ovipositing mosquitoes. A removable capture chamber above the pail features a funnel entrance and an inner lining coated with polybutylene adhesive (32UVR, Atlantic Paste & Glue Co., Brooklyn, NY) to trap and immobilize mosquitoes. A mesh screen separates the infusion from the chamber, allowing volatile odors to escape while preventing access to liquid.

Three traps were installed per household in shaded outdoor areas (e.g., patios), targeting preferred *Ae. aegypti* resting and oviposition sites. Deployment began in December 2011 in La Margarita and February 2013 in Villodas, and ultimately covered ~85% of households in each intervention community. Households were neither included nor excluded from participation based on trap presence.

Traps were installed and serviced by trained field staff from the CDC Dengue Branch in collaboration with local municipal workers (*4*). All staff underwent standardized training in AGO assembly, placement, and servicing, and followed written protocols to ensure uniform delivery across households. Traps remained in place between service visits, which occurred every 2 months. During each visit, staff replenished water, replaced hay packets, and applied

fresh adhesive. This servicing schedule, validated in long-term entomologic trials, sustained consistent capture rates throughout each 8-week cycle (*2*,*5*).

To achieve and maintain high coverage, community engagement was emphasized from the outset. Teams conducted door-to-door visits, explained the purpose of the intervention, and provided educational materials on mosquito control. Consent was obtained at the household level, and informational materials were provided in Spanish. Although participation was voluntary and no financial incentives were offered, community acceptance was high. Households that declined participation were revisited periodically to encourage enrollment, and replacement traps were installed if devices were damaged or removed. In addition, feedback loops were maintained by providing households with information about arbovirus risks and, in other related studies, with individual or household-level results, which helped foster sustained trust and cooperation. These strategies, combined with consistent servicing and rapport with residents, enabled sustained coverage of ~85% of households over multiple years (*4*).

**Participant Data Collection**

Data were collected through two structured questionnaires: a household-level form completed by the head of household and an individual-level form completed by or on behalf of each participant. The household questionnaire captured dwelling characteristics (e.g., structure type, air conditioning, screened windows/doors), household composition, income, mosquito prevention behaviors (e.g., coils, citronella), and—among intervention households—perceptions of AGO effectiveness.

The individual questionnaire gathered demographic information (age, sex, pregnancy status), duration of residence, ZIKV-like illness history since November 2015 (rash, fever, joint pain), and healthcare use. Participants also reported prevention behaviors (e.g., repellent, bed net use), mosquito bite frequency and location (home versus elsewhere), and hours spent at home or in the community by day. These estimates were summed to calculate weekly time-at-home, which was categorized into tertiles. For children aged 5–8 years, a parent or guardian provided responses. All interviews were conducted in Spanish by trained staff following standardized protocols. Serum specimens were labeled with participant IDs to maintain traceability and data integrity.

### Entomological Surveillance

For entomologic surveillance, sentinel AGOs (SAGO), identical in design but used for entomologic surveillance, were also deployed at fixed intervals across all four communities and checked weekly (*1*,*2*,*6*). SAGOs were placed systematically across neighborhoods at fixed points to minimize trap interaction and provide representative measures of mosquito populations. These traps provided real-time measures of mosquito abundance and infection rates and confirmed that differences between intervention and nonintervention areas only emerged after AGO implementation. No insecticides were used at any time.

In addition to abundance monitoring, mosquito pools collected during 2016 were tested for ZIKV, CHIKV, and DENV RNA using trioplex RT-PCR as reported previously (*5*). ZIKV was detected in multiple pools from nonintervention communities but only rarely in intervention areas, consistent with entomological evidence of reduced transmission risk.

To evaluate temporal associations between mosquito abundance and ZIKV infection, each participant was linked to weekly *Ae. aegypti* surveillance data from their community. Lagged mosquito abundance values (0–2 weeks before testing) were derived from AGO counts and aligned with each participant's week of enrollment.

### Statistical Analysis

We compared demographic, household, and behavioral characteristics of participants in intervention and nonintervention communities using Pearson's chi-square test for categorical variables, Fisher exact test for sparse data, and the Mann–Whitney U test for nonnormally distributed continuous variables. The primary analysis included participants positive for anti-ZIKV IgM and negative for anti-DENV IgM to reduce cross-reactivity. Seroprevalence was estimated overall, by intervention status, and across key demographic and household subgroups.

To assess the association between intervention status and ZIKV infection, we used Poisson regression with a log link and robust standard errors to estimate prevalence ratios (PRs) and 95% confidence intervals (CIs) (*7*). Robust errors were calculated using the Huber-White sandwich estimator to account for model misspecification (*8*). Multivariable models adjusted for covariates selected a priori based on existing literature and biologic plausibility, including age group, sex, and time spent at home during daylight hours, to account for differential mosquito exposure risk (*9*). Effect modification was explored by stratifying models by age group, sex,

household size, air conditioning, window screening, mosquito bite frequency, use of repellent or bed nets, and window-opening behavior. We also used generalized linear models with a binomial link and cubic splines to estimate the predicted probability of ZIKV seropositivity by weekly hours spent at home, stratified by intervention status. As a sensitivity analysis, we repeated all models using a broader outcome—ZIKV and/or DENV IgM seropositivity—to capture overall arboviral exposure. This approach accounts for shared transmission pathways and potential IgM cross-reactivity. Prevalence ratios were re-estimated using the same model framework and covariates.

To examine associations between *Ae. aegypti* female abundance and ZIKV IgM seropositivity, we fit Poisson regression models with a log link and robust (sandwich) standard errors. Weekly mosquito abundance was modeled as a continuous predictor (per +1 female per trap-week) and evaluated at lags of 0–2 weeks relative to specimen collection (lag 0 = same week; lags 1–2 = 1–2 weeks prior). We estimated pooled associations in the full sample and formally assessed effect modification by community intervention status by including a mosquito abundance * intervention interaction term in pooled models; interaction was evaluated using Wald tests based on robust standard errors. For descriptive purposes, we report community-specific risk ratios (RRs) for intervention and nonintervention communities estimated from the pooled interaction model (i.e., using a single model with a mosquito abundance * intervention term), rather than fitting separate models within each community type. Adjusted models additionally included age category, sex, and hours spent at home (community) category. RRs with 95% confidence intervals (CIs) are reported. Analyses were conducted in R version 4.4.2 (The R Project for Statistical Computing, https://www.r-project.org), using two-sided tests with $p<0.05$ considered statistically significant.

**References**

1. Barrera R, Amador M, Acevedo V, Caban B, Felix G, Mackay AJ. Use of the CDC autocidal gravid ovitrap to control and prevent outbreaks of *Aedes aegypti* (Diptera: Culicidae). J Med Entomol. 2014;51:145–54. PubMed https://doi.org/10.1603/ME13096

2. Barrera R, Amador M, Acevedo V, Hemme RR, Félix G. Sustained, area-wide control of *Aedes aegypti* using CDC autocidal gravid ovitraps. Am J Trop Med Hyg. 2014;91:1269–76. PubMed https://doi.org/10.4269/ajtmh.14-0426

3. Mackay AJ, Amador M, Barrera R. An improved autocidal gravid ovitrap for the control and surveillance of *Aedes aegypti*. Parasit Vectors. 2013;6:225. PubMed https://doi.org/10.1186/1756-3305-6-225

4. Hemme RR, Smith EA, Felix G, White BJ, Diaz-Garcia MI, Rodriguez D, et al. Multi-year mass-trapping with autocidal gravid ovitraps has limited influence on insecticide susceptibility in *Aedes aegypti* (Diptera: Culicidae) from Puerto Rico. J Med Entomol. 2022;59:314–9. PubMed https://doi.org/10.1093/jme/tjab162

5. Barrera R, Amador M, Acevedo V, Beltran M, Muñoz JL. A comparison of mosquito densities, weather and infection rates of *Aedes aegypti* during the first epidemics of chikungunya (2014) and Zika (2016) in areas with and without vector control in Puerto Rico. Med Vet Entomol. 2019;33:68–77. PubMed https://doi.org/10.1111/mve.12338

6. Madewell ZJ, Hemme RR, Adams L, Barrera R, Waterman SH, Johansson MA. Comparing vector and human surveillance strategies to detect arbovirus transmission: a simulation study for Zika virus detection in Puerto Rico. PLoS Negl Trop Dis. 2019;13:e0007988. PubMed https://doi.org/10.1371/journal.pntd.0007988

7. Zou G. A modified Poisson regression approach to prospective studies with binary data. Am J Epidemiol. 2004;159:702–6. PubMed https://doi.org/10.1093/aje/kwh090

8. Zeileis A. sandwich: robust covariance matrix estimators. 2024 [cited 2025 Apr 11]. https://cran.r-project.org/web/packages/sandwich/index.html

9. Sharp TM, Lorenzi O, Torres-Velásquez B, Acevedo V, Pérez-Padilla J, Rivera A, et al. Autocidal gravid ovitraps protect humans from chikungunya virus infection by reducing Aedes aegypti mosquito populations. PLoS Negl Trop Dis. 2019;13:e0007538. PubMed https://doi.org/10.1371/journal.pntd.0007538

**Appendix Table 1.** Baseline demographic, geographic, and household characteristics of study communities, Salinas, Puerto Rico.

| | Intervention | | Nonintervention | |
|---|---|---|---|---|
| Characteristic | La Margarita | Villodas | La Playa | Arboleda |
| Municipality | Salinas | Guayama | Salinas | Guayama |
| Elevation, m | 3 | 20 | 10 | 1 |
| Area, hectares | 18 | 11 | 17 | 21 |
| Estimated 2010 population | 579 | 639 | 484 | 800 |
| Population density/hectare | 32.2 | 58.1 | 28.5 | 38.1 |
| No. residential structures | 327 | 235 | 273 | 393 |
| % Occupied households | 85.0 | 77.4 | 73.3 | 90.1 |
| Average household size, no. persons | 2.6 | 3.6 | 2.6 | 2.7 |
| Predominant language | Spanish | Spanish | Spanish | Spanish |

**Appendix Table 2.** Demographic characteristics and reported behaviors among participants of a survey of Zika virus seroprevalence among communities with (intervention) or without (nonintervention) autocidal gravid ovitraps (AGOs) in Puerto Rico, March – May 2017.

| Characteristics | Total no. (%), n = 271 | No. (%) participants: Intervention, n = 136 | No. (%) participants: Nonintervention, n = 135 | p value† |
|---|---|---|---|---|
| Age group, y; n = 267 | | | | 0.283 |
| <20 | 18 (6.7) | 9 (6.7) | 9 (6.8) | |
| 20–39 | 31 (11.6) | 19 (14.2) | 12 (9.0) | |
| 40–59 | 86 (32.2) | 47 (35.1) | 39 (29.3) | |
| ≥60 | 132 (49.4) | 59 (44.0) | 73 (54.9) | |
| Median age, y (IQR) | 59 (46–69) | 59 (43–69) | 60 (49–69) | 0.324 |
| Sex | | | | 0.242 |
| F | 165 (60.9) | 88 (64.7) | 77 (57.0) | |
| M | 106 (39.1) | 48 (35.3) | 58 (43.0) | |
| Years in community, n = 269 | | | | 0.244 |
| 0–19 | 104 (38.7) | 56 (41.2) | 48 (36.1) | |
| 20–39 | 116 (43.1) | 52 (38.2) | 64 (48.1) | |
| ≥40 | 49 (18.2) | 28 (20.6) | 21 (15.8) | |
| Median years (IQR) in community, n = 269 | 24 (12–35) | 21 (13–35) | 29 (12–34) | 0.307 |
| Acute illness with rash, fever, or joint pain since November 2015, n = 270 | | | | 0.271 |
| Y | 58 (21.5) | 25 (18.4) | 33 (24.6) | |
| N | 212 (78.5) | 111 (81.6) | 101 (75.4) | |
| Household characteristics | | | | |
| No. persons in household | | | | 0.516 |
| 1 | 60 (22.1) | 30 (22.1) | 30 (22.2) | |
| 2 | 101 (37.3) | 47 (34.6) | 54 (40.0) | |
| 3 | 52 (19.2) | 25 (18.4) | 27 (20.0) | |
| ≥4 | 58 (21.4) | 34 (25.0) | 24 (17.8) | |
| Median no. (IQR) persons in household | 2 (2–3) | 2 (2–3) | 2 (2–3) | 0.454 |
| No. children <5 years of age | | | | 0.081 |
| 0 | 250 (92.3) | 121 (89.0) | 129 (95.6) | |
| 1 | 17 (6.3) | 13 (9.6) | 4 (3.0) | |
| 2 | 4 (1.5) | 2 (1.5) | 2 (1.5) | |
| Annual household income, USD; n = 238 | | | | 0.059 |
| <25,000 | 173 (72.7) | 88 (73.3) | 85 (72.0) | |
| 26,000–50,000 | 46 (19.3) | 18 (15.0) | 28 (23.7) | |
| 51,000–75,000 | 16 (6.7) | 11 (9.2) | 5 (4.2) | |
| >76,000 | 3 (1.3) | 3 (2.5) | 0 (0.0) | |
| Dwelling type | | | | 0.393 |
| One-story house | 248 (91.5) | 122 (89.7) | 126 (93.3) | |
| Two-story house | 23 (8.5) | 14 (10.3) | 9 (6.7) | |
| No. screened windows, doors; n = 270 | | | | 0.789 |
| 1 | 139 (51.5) | 70 (51.9) | 69 (51.1) | |
| 2 | 87 (32.2) | 45 (33.3) | 42 (31.1) | |
| 3 | 44 (16.3) | 20 (14.8) | 24 (17.8) | |
| Air conditioning use | | | | 0.312 |
| In all rooms | 42 (15.5) | 18 (13.2) | 24 (17.8) | |
| Only in bedroom at night | 140 (51.7) | 68 (50.0) | 72 (53.3) | |
| None | 89 (32.8) | 50 (36.8) | 39 (28.9) | |
| Frequency of open windows, doors | | | | 0.496 |
| Always | 96 (35.4) | 46 (33.8) | 50 (37.0) | |
| Only during the day | 116 (42.8) | 63 (46.3) | 53 (39.3) | |
| Only at night | 7 (2.6) | 2 (1.5) | 5 (3.7) | |
| Never | 52 (19.2) | 25 (18.4) | 27 (20.0) | |
| Mosquito exposure | | | | |
| Weekly hours at home | | | | 0.827 |
| ≤24 | 19 (7.0) | 9 (6.6) | 10 (7.4) | |
| 25–60 | 91 (33.6) | 48 (35.3) | 43 (31.9) | |
| 61–84 | 161 (59.4) | 79 (58.1) | 82 (60.7) | |
| Median weekly hours (IQR) at home | 73 (42–84) | 72 (42–84) | 74 (44–84) | 0.536 |
| Frequency of mosquito bites, n = 259 | | | | 0.004 |
| Daily | 44 (16.3) | 11 (8.1) | 33 (24.6) | |
| Weekly | 40 (14.8) | 19 (14.0) | 21 (15.7) | |
| Rarely | 154 (57.0) | 86 (63.2) | 68 (50.7) | |

| Characteristics | Total no. (%), n = 271 | No. (%) participants Intervention, n = 136 | Nonintervention, n = 135 | p value† |
|---|---|---|---|---|
| Never | 21 (7.8) | 14 (10.3) | 7 (5.2) | |
| Time of mosquito bites‡ | | | | |
| Morning | 36 (13.3) | 14 (10.3) | 22 (16.3) | 0.202 |
| Daytime | 30 (11.1) | 14 (10.3) | 16 (11.9) | 0.830 |
| Afternoon | 157 (57.9) | 72 (52.9) | 85 (63.0) | 0.122 |
| Night | 95 (35.1) | 53 (39.0) | 42 (31.1) | 0.219 |
| Never | 21 (7.7) | 14 (10.3) | 7 (5.2) | 0.178 |
| Location of mosquito bites‡ | | | | |
| Home | 198 (73.1) | 91 (66.9) | 107 (79.3) | 0.031 |
| School or work | 25 (9.2) | 14 (10.3) | 11 (8.1) | 0.689 |
| Other houses in this community | 30 (11.1) | 17 (12.5) | 13 (9.6) | 0.576 |
| Other houses outside of this community | 32 (11.8) | 18 (13.2) | 14 (10.4) | 0.587 |
| Other places | 42 (15.5) | 21 (15.4) | 21 (15.6) | 1 |
| Mosquito prevention behaviors | | | | |
| Frequency of repellent use, n = 270 | | | | 0.474 |
| Daily | 41 (15.2) | 17 (12.5) | 24 (17.9) | |
| Weekly | 31 (11.5) | 13 (9.6) | 18 (13.4) | |
| Monthly | 14 (5.2) | 8 (5.9) | 6 (4.5) | |
| Rarely | 115 (42.6) | 59 (43.4) | 56 (41.8) | |
| Never | 69 (25.6) | 39 (28.7) | 30 (22.4) | |
| Frequency of net use, n = 270 | | | | 0.123 |
| Daily | 5 (1.9) | 2 (1.5) | 3 (2.2) | |
| Rarely | 4 (1.5) | 4 (2.9) | 0 (0.0) | |
| Never | 261 (96.7) | 130 (95.6) | 131 (97.8) | |
| Use of mosquito coils, n = 269 | | | | 0.967 |
| Y | 64 (23.8) | 33 (24.3) | 31 (23.3) | |
| N | 205 (76.2) | 103 (75.7) | 102 (76.7) | |
| Use of citronella | | | | 0.004 |
| Y | 65 (24.0) | 22 (16.2) | 43 (31.9) | |
| N | 206 (76.0) | 114 (83.8) | 92 (68.1) | |

*IQR, interquartile range.
†$\chi^2$ test was used for comparison of proportions, Fisher exact test was used for comparison of proportions with at least 1 value <5, and Mann-Whitney U test was used for medians.
‡Time and location of mosquito bites were select-all-that-apply items; percentages might not sum to 100%.

**Appendix Table 3.** Comparison of demographic characteristics and reported behaviors among participants with valid ZIKV or DENV IgM results from communities with (intervention) or without (nonintervention) autocidal gravid ovitraps (AGOs) in Puerto Rico, March – May 2017.

| Characteristic | Total no. (%), n = 297 | No. (%) participants: Intervention, n = 153 | No. (%) participants: Nonintervention, n = 144 | p value† |
|---|---|---|---|---|
| Participant demographic characteristics | | | | |
| Age group, y; n = 293 | | | | 0.414 |
| <20 | 20 (6.8) | 11 (7.3) | 9 (6.3) | |
| 20–39 | 33 (11.3) | 20 (13.2) | 13 (9.2) | |
| 40–59 | 89 (30.4) | 49 (32.5) | 40 (28.2) | |
| >60 | 151 (51.5) | 71 (47.0) | 80 (56.3) | |
| Median age [IQR] | 60 [46, 69] | 59 [43, 70] | 60 [49, 69] | 0.437 |
| Sex | | | | 0.258 |
| M | 115 (38.7) | 54 (35.3) | 61 (42.4) | |
| F | 182 (61.3) | 99 (64.7) | 83 (57.6) | |
| Pregnant, n = 34‡ | | | | 1.000 |
| Y | 1 (2.9) | 1 (4.3) | 0 (0.0) | |
| N | 33 (97.1) | 22 (95.7) | 11 (100.0) | |
| Years residing in the community, n = 295 | | | | 0.144 |
| 0–19 | 112 (38.0) | 63 (41.2) | 49 (34.5) | |
| 20–39 | 124 (42.0) | 56 (36.6) | 68 (47.9) | |
| >40 | 59 (20.0) | 34 (22.2) | 25 (17.6) | |
| Median years in community [IQR] | 25 [13, 35] | 21 [13, 37] | 30 [13, 35] | 0.244 |
| Had acute illness with rash, fever, or joint pain since November 2015 | | | | 0.150 |
| Y | 63 (21.3) | 27 (17.6) | 36 (25.2) | |
| N | 233 (78.7) | 126 (82.4) | 107 (74.8) | |
| Household characteristics | | | | |
| Household size, no. persons | | | | 0.600 |
| 1 | 66 (22.2) | 34 (22.2) | 32 (22.2) | |
| 2 | 111 (37.4) | 54 (35.3) | 57 (39.6) | |
| 3 | 59 (19.9) | 29 (19.0) | 30 (20.8) | |
| >4 | 61 (20.5) | 36 (23.5) | 25 (17.4) | |
| Median household size [IQR] | 2 [2, 3] | 2 [2, 3] | 2 [2, 3] | 0.541 |
| No. children <5 years of age in household | | | | 0.071 |
| 0 | 275 (92.6) | 137 (89.5) | 138 (95.8) | |
| 1 | 18 (6.1) | 14 (9.2) | 4 (2.8) | |
| 2 | 4 (1.3) | 2 (1.3) | 2 (1.4) | |
| Annual household income, USD; n = 261 | | | | 0.158 |
| <$25,000 | 190 (72.8) | 99 (73.3) | 91 (72.2) | |
| $26,000 – $50,000 | 50 (19.2) | 21 (15.6) | 29 (23.0) | |
| $51,000 – $75,000 | 17 (6.5) | 12 (8.9) | 5 (4.0) | |
| >$76,000 | 4 (1.5) | 3 (2.2) | 1 (0.8) | |
| Dwelling type | | | | 0.363 |
| One-story house | 273 (91.9) | 138 (90.2) | 135 (93.8) | |
| Two-story house | 24 (8.1) | 15 (9.8) | 9 (6.2) | |
| No. screened windows/doors, n = 296 | | | | 0.917 |
| 1 | 154 (52.0) | 79 (52.0) | 75 (52.1) | |
| 2 | 95 (32.1) | 50 (32.9) | 45 (31.2) | |
| 3 | 47 (15.9) | 23 (15.1) | 24 (16.7) | |
| Air conditioning use | | | | 0.385 |
| In all rooms | 47 (15.8) | 22 (14.4) | 25 (17.4) | |
| Only in bedroom at night | 150 (50.5) | 74 (48.4) | 76 (52.8) | |
| No | 100 (33.7) | 57 (37.3) | 43 (29.9) | |
| Frequency of open windows/doors | | | | 0.735 |
| Always | 102 (34.3) | 50 (32.7) | 52 (36.1) | |
| Only during the day | 129 (43.4) | 70 (45.8) | 59 (41.0) | |
| Only at night | 8 (2.7) | 3 (2.0) | 5 (3.5) | |
| Never | 58 (19.5) | 30 (19.6) | 28 (19.4) | |
| Mosquito Exposure | | | | |
| Weekly hours at home | | | | 0.671 |
| ≤24 | 21 (7.1) | 11 (7.2) | 10 (6.9) | |
| 25 – 60 | 100 (33.7) | 55 (35.9) | 45 (31.2) | |
| 61 – 84 | 176 (59.3) | 87 (56.9) | 89 (61.8) | |
| Median weekly hours at home [IQR] | 72 [42, 84] | 70 [42, 84] | 74 [44, 84] | 0.329 |
| Frequency of mosquito bites | | | | 0.019 |
| Daily | 50 (16.9) | 16 (10.5) | 34 (23.8) | |
| Weekly | 45 (15.2) | 21 (13.7) | 24 (16.8) | |

| Characteristic | Total no. (%), n = 297 | No. (%) participants | | p value† |
|---|---|---|---|---|
| | | Intervention, n = 153 | Nonintervention, n = 144 | |
| Rarely | 166 (56.1) | 94 (61.4) | 72 (50.3) | |
| Never | 23 (7.8) | 15 (9.8) | 8 (5.6) | |
| Time of mosquito bites | | | | |
| Morning | 40 (13.5) | 17 (11.1) | 23 (16.0) | 0.291 |
| Daytime | 33 (11.1) | 15 (9.8) | 18 (12.5) | 0.579 |
| Afternoon | 175 (58.9) | 84 (54.9) | 91 (63.2) | 0.182 |
| Night | 102 (34.3) | 57 (37.3) | 45 (31.2) | 0.334 |
| Never | 23 (7.7) | 15 (9.8) | 8 (5.6) | 0.249 |
| Location of mosquito bites | | | | |
| Home | 216 (72.7) | 104 (68.0) | 112 (77.8) | 0.077 |
| School or work | 26 (8.8) | 15 (9.8) | 11 (7.6) | 0.650 |
| Other houses in this community | 33 (11.1) | 18 (11.8) | 15 (10.4) | 0.853 |
| Other houses outside of this community | 36 (12.1) | 18 (11.8) | 18 (12.5) | 0.987 |
| Other places | 48 (16.2) | 25 (16.3) | 23 (16.0) | 1 |
| Mosquitoes never bite me | 16 (5.4) | 10 (6.5) | 6 (4.2) | 0.518 |
| Mosquito Prevention Behaviors | | | | |
| Frequency of repellent use | | | | 0.351 |
| Daily | 46 (15.5) | 21 (13.7) | 25 (17.5) | |
| Weekly | 33 (11.1) | 13 (8.5) | 20 (14.0) | |
| Monthly | 15 (5.1) | 8 (5.2) | 7 (4.9) | |
| Rarely | 126 (42.6) | 66 (43.1) | 60 (42.0) | |
| Never | 76 (25.7) | 45 (29.4) | 31 (21.7) | |
| Frequency of net use | | | | 0.133 |
| Daily | 5 (1.7) | 2 (1.3) | 3 (2.1) | |
| Rarely | 4 (1.4) | 4 (2.6) | 0 (0.0) | |
| Never | 287 (97.0) | 147 (96.1) | 140 (97.9) | |
| Use of mosquito coils | | | | 0.949 |
| Y | 68 (23.1) | 36 (23.5) | 32 (22.5) | |
| N | 227 (76.9) | 117 (76.5) | 110 (77.5) | |
| Use of citronella | | | | 0.014 |
| Y | 73 (24.6) | 28 (18.3) | 45 (31.2) | |
| N | 224 (75.4) | 125 (81.7) | 99 (68.8) | |
| IgM Test Results | | | | |
| DENV IgM result | | | | 0.237 |
| Positive | 5 (1.7) | 1 (0.7) | 4 (2.8) | |
| Negative | 286 (96.3) | 150 (98.0) | 136 (94.4) | |
| Equivocal/Indeterminate | 6 (2.0) | 2 (1.3) | 4 (2.8) | |
| ZIKV IgM result | | | | 0.012 |
| Positive | 41 (13.8) | 13 (8.5) | 28 (19.4) | |
| Negative | 234 (78.8) | 124 (81.0) | 110 (76.4) | |
| Equivocal/Indeterminate | 16 (5.4) | 11 (7.2) | 5 (3.5) | |
| Uninterpretable (high background) | 6 (2.0) | 5 (3.3) | 1 (0.7) | |

*Values are no. (%) except as indicated. DENV, dengue virus; ZIKV, Zika virus.

†$\chi^2$ test was used for comparison of proportions, Fisher exact test was used for comparison of proportions with at least 1 value <5, and Mann-Whitney U test was used for medians.

‡Denominators for these analyses include visits from women of childbearing age defined as any female aged 15–44 y.

**Appendix Table 4.** Zika virus IgM seropositivity among residents with valid serologic results in communities with and without autocidal gravid ovitraps (AGOs), by demographic and behavioral characteristics, Puerto Rico, March – May 2017

| Characteristic | % ZIKV IgM seropositivity (no. positive/no. tested)† Intervention, n = 136 | Nonintervention, n = 135 | PR (95% CI) | aPR (95% CI)‡ |
|---|---|---|---|---|
| Overall | 9.6 (13/136) | 20.0 (27/135) | 0.48 (0.26–0.89) | 0.49 (0.27–0.90) |
| Age group, y | | | | |
| <20 | 0.0 (0/9) | 22.2 (2/9) | 0.00 (0.00–Inf) | 0.00 (0.00–Inf) |
| 20–39 | 31.6 (6/19) | 16.7 (2/12) | 1.89 (0.38–9.39) | 1.93 (0.39–9.63) |
| 40–59 | 8.5 (4/47) | 15.4 (6/39) | 0.55 (0.16–1.96) | 0.55 (0.16–1.95) |
| ≥60 | 5.1 (3/59) | 23.3 (17/73) | 0.22 (0.06–0.75) | 0.22 (0.06–0.75) |
| Sex | | | | |
| F | 8 (7/88) | 16.9 (13/77) | 0.47 (0.19–1.18) | 0.47 (0.19–1.18) |
| M | 12.5 (6/48) | 24.1 (14/58) | 0.52 (0.20–1.35) | 0.52 (0.20–1.35) |
| Household size, no. persons | | | | |
| 1 | 10 (3/30) | 20 (6/30) | 0.50 (0.13–2.00) | 0.56 (0.14–2.24) |
| 2 | 14.9 (7/47) | 13 (7/54) | 1.15 (0.40–3.28) | 1.11 (0.39–3.19) |
| 3 | 8 (2/25) | 25.9 (7/27) | 0.31 (0.06–1.49) | 0.33 (0.07–1.58) |
| ≥4 | 2.9 (1/34) | 29.2 (7/24) | 0.10 (0.01–0.82) | 0.10 (0.01–0.81) |
| Dwelling type | | | | |
| One-story house | 9.8 (12/122) | 19 (24/126) | 0.52 (0.26–1.03) | 0.52 (0.26–1.03) |
| Two-story house | 7.1 (1/14) | 33.3 (3/9) | 0.21 (0.02–2.06) | 0.21 (0.02–2.06) |
| Number of screened windows/doors | | | | |
| 1 | 8.6 (6/70) | 14.5 (10/69) | 0.59 (0.21–1.63) | 0.59 (0.21–1.61) |
| 2 | 8.9 (4/45) | 23.8 (10/42) | 0.37 (0.12–1.19) | 0.36 (0.11–1.15) |
| 3 | 15 (3/20) | 29.2 (7/24) | 0.51 (0.13–1.99) | 0.44 (0.11–1.72) |
| Air conditioning use | | | | |
| In all rooms | 11.1 (2/18) | 12.5 (3/24) | 0.89 (0.15–5.32) | 1.02 (0.17–6.27) |
| Only in bedroom at night | 8.8 (6/68) | 13.9 (10/72) | 0.64 (0.23–1.75) | 0.64 (0.23–1.75) |
| No | 10 (5/50) | 35.9 (14/39) | 0.28 (0.10–0.77) | 0.28 (0.10–0.78) |
| Frequency of open windows/doors | | | | |
| Always | 6.5 (3/46) | 24 (12/50) | 0.27 (0.08–0.96) | 0.27 (0.08–0.97) |
| Only during the day | 14.3 (9/63) | 18.9 (10/53) | 0.76 (0.31–1.86) | 0.75 (0.30–1.85) |
| Never | 4 (1/25) | 18.5 (5/27) | 0.22 (0.03–1.85) | 0.22 (0.03–1.92) |
| Weekly hours at home§ | | | | |
| ≤24 | 11.1 (1/9) | 10.0 (1/10) | 1.11 (0.07–17.76) | – |
| 25 – 60 | 16.7 (8/48) | 18.6 (8/43) | 0.90 (0.34–2.39) | – |
| 61 – 84 | 5.1 (4/79) | 22.0 (18/82) | 0.23 (0.08–0.68) | – |
| Frequency of mosquito bites | | | | |
| Daily | 9.1 (1/11) | 27.3 (9/33) | 0.33 (0.04–2.63) | 0.34 (0.04–2.72) |
| Weekly | 0 (0/19) | 19 (4/21) | 0.00 (0.00–Inf) | 0.00 (0.00–Inf) |
| Rarely | 11.6 (10/86) | 19.1 (13/68) | 0.61 (0.27–1.39) | 0.60 (0.26–1.37) |
| Never | 14.3 (2/14) | 14.3 (1/7) | 1.00 (0.09–11.03) | 1.69 (0.11–27.23) |
| Frequency of repellent use | | | | |
| Daily | 23.5 (4/17) | 29.2 (7/24) | 0.81 (0.24–2.76) | 0.79 (0.23–2.71) |
| Weekly | 7.7 (1/13) | 16.7 (3/18) | 0.46 (0.05–4.44) | 0.45 (0.05–4.35) |
| Monthly | 0 (0/8) | 33.3 (2/6) | 0.00 (0.00–Inf) | 0.00 (0.00–Inf) |
| Rarely | 5.1 (3/59) | 14.3 (8/56) | 0.36 (0.09–1.34) | 0.37 (0.10–1.39) |
| Never | 12.8 (5/39) | 23.3 (7/30) | 0.55 (0.17–1.73) | 0.56 (0.18–1.78) |
| Frequency of net use | | | | |
| Daily | 0 (0/2) | 100 (3/3) | 0.00 (0.00–Inf) | 0.00 (0.00–Inf) |
| Rarely | 0 (0/4) | – | – | – |
| Never | 10.0 (13/130) | 18.3 (24/131) | 0.55 (0.28–1.07) | 0.54 (0.28–1.07) |
| Use of mosquito coils | | | | |
| Yes | 6.1 (2/33) | 35.5 (11/31) | 0.17 (0.04–0.77) | 0.17 (0.04–0.78) |
| No | 10.7 (11/103) | 15.7 (16/102) | 0.68 (0.32–1.47) | 0.68 (0.32–1.47) |
| Use of citronella | | | | |
| Yes | 4.5 (1/22) | 23.3 (10/43) | 0.20 (0.03–1.53) | 0.20 (0.03–1.55) |
| No | 10.5 (12/114) | 18.5 (17/92) | 0.57 (0.27–1.19) | 0.57 (0.27–1.19) |

*aPR adjusted prevalence ratio; PR, prevalence ratio; ZIKV, Zika virus; –, not applicable.
†Column n indicates the analytic sample with valid serologic results (interpretable ZIKV IgM and DENV IgM negative). Cell denominators (No.) reflect participants with nonmissing data for each characteristic.
‡All models were adjusted for time spent at home during daylight hours, except the overall model, which was additionally adjusted for age group and sex. Adjustment approach is consistent with Sharp et al. (*9*).
§Adjusted PRs are not presented for *weekly hours at home* because this variable was used as an adjustment covariate in other models.

**Appendix Table 5.** Zika virus and/or dengue virus IgM seropositivity among residents with valid serologic results in communities with and without autocidal gravid ovitraps (AGOs), by demographic and behavioral characteristics, Puerto Rico, March – May 2017.

| Characteristic | % ZIKV and/or DENV IgM seropositivity (no. positive/no. tested)† Intervention, n = 153 | % ZIKV and/or DENV IgM seropositivity (no. positive/no. tested)† Nonintervention, n = 144 | PR (95% CI) | aPR (95% CI)‡ |
|---|---|---|---|---|
| Overall | 9.2 (14/153) | 21.5 (31/144) | 0.43 (0.24–0.77) | 0.44 (0.24–0.78) |
| Age group, y | | | | |
| <20 | 0 (0/11) | 22.2 (2/9) | 0.00 (0.00–Inf) | 0.00 (0.00–Inf) |
| 20–39 | 30 (6/20) | 15.4 (2/13) | 1.95 (0.39–9.66) | 1.98 (0.40–9.88) |
| 40–59 | 8.2 (4/49) | 15 (6/40) | 0.54 (0.15–1.93) | 0.54 (0.15–1.92) |
| ≥60 | 5.6 (4/71) | 26.2 (21/80) | 0.21 (0.07–0.63) | 0.21 (0.07–0.62) |
| Sex | | | | |
| F | 8.1 (8/99) | 18.1 (15/83) | 0.45 (0.19–1.05) | 0.45 (0.19–1.07) |
| M | 11.1 (6/54) | 26.2 (16/61) | 0.42 (0.17–1.08) | 0.42 (0.17–1.08) |
| Household size, no. persons | | | | |
| 1 | 11.8 (4/34) | 21.9 (7/32) | 0.54 (0.16–1.84) | 0.58 (0.17–1.99) |
| 2 | 13 (7/54) | 15.8 (9/57) | 0.82 (0.31–2.20) | 0.81 (0.30–2.20) |
| 3 | 6.9 (2/29) | 26.7 (8/30) | 0.26 (0.05–1.22) | 0.27 (0.06–1.26) |
| ≥4 | 2.8 (1/36) | 28 (7/25) | 0.10 (0.01–0.81) | 0.10 (0.01–0.80) |
| Dwelling type | | | | |
| One-story house | 9.4 (13/138) | 20.7 (28/135) | 0.45 (0.24–0.88) | 0.45 (0.24–0.88) |
| Two-story house | 6.7 (1/15) | 33.3 (3/9) | 0.20 (0.02–1.92) | 0.20 (0.02–1.92) |
| Number of screened windows/doors | | | | |
| 1 | 7.6 (6/79) | 17.3 (13/75) | 0.44 (0.17–1.15) | 0.43 (0.16–1.14) |
| 2 | 8.0 (4/50) | 24.4 (11/45) | 0.33 (0.10–1.03) | 0.32 (0.10–1.00) |
| 3 | 17.4 (4/23) | 29.2 (7/24) | 0.60 (0.17–2.04) | 0.50 (0.14–1.74) |
| Air conditioning use | | | | |
| In all rooms | 9.1 (2/22) | 16.0 (4/25) | 0.57 (0.10–3.10) | 0.56 (0.10–3.06) |
| Only in bedroom at night | 9.5 (7/74) | 15.8 (12/76) | 0.60 (0.24–1.52) | 0.61 (0.24–1.55) |
| No | 8.8 (5/57) | 34.9 (15/43) | 0.25 (0.09–0.69) | 0.25 (0.09–0.69) |
| Frequency of open windows/doors | | | | |
| Always | 8.0 (4/50) | 23.1 (12/52) | 0.35 (0.11–1.07) | 0.35 (0.11–1.08) |
| Only during the day | 12.9 (9/70) | 22 (13/59) | 0.58 (0.25–1.37) | 0.59 (0.25–1.39) |
| Never | 3.3 (1/30) | 21.4 (6/28) | 0.16 (0.02–1.29) | 0.16 (0.02–1.34) |
| Weekly hours at home§ | | | | |
| ≤24 | 9.1 (1/11) | 10.0 (1/10) | 0.91 (0.06–14.53) | – |
| 25 – 60 | 14.5 (8/55) | 20.0 (9/45) | 0.73 (0.28–1.88) | – |
| 61 – 84 | 5.7 (5/87) | 23.6 (21/89) | 0.24 (0.09–0.65) | – |
| Frequency of mosquito bites | | | | |
| Daily | 6.2 (1/16) | 26.5 (9/34) | 0.24 (0.03–1.86) | 0.25 (0.03–2.05) |
| Weekly | 4.8 (1/21) | 20.8 (5/24) | 0.23 (0.03–1.96) | 0.24 (0.03–2.02) |
| Rarely | 10.6 (10/94) | 20.8 (15/72) | 0.51 (0.23–1.14) | 0.51 (0.23–1.13) |
| Never | 13.3 (2/15) | 25 (2/8) | 0.53 (0.08–3.79) | 0.62 (0.08–4.96) |
| Frequency of repellent use | | | | |
| Daily | 19 (4/21) | 28.0 (7/25) | 0.68 (0.20–2.32) | 0.58 (0.17–2.00) |
| Weekly | 7.7 (1/13) | 25.0 (5/20) | 0.31 (0.04–2.63) | 0.30 (0.03–2.58) |
| Monthly | 0 (0/8) | 28.6 (2/7) | 0.00 (0.00–Inf) | 0.00 (0.00–Inf) |
| Rarely | 4.5 (3/66) | 16.7 (10/60) | 0.27 (0.08–0.99) | 0.29 (0.08–1.05) |
| Never | 13.3 (6/45) | 22.6 (7/31) | 0.59 (0.20–1.76) | 0.62 (0.21–1.84) |
| Frequency of net use | | | | |
| Daily | 0 (0/2) | 100 (3/3) | 0.00 (0.00–Inf) | 0.00 (0.00–Inf) |
| Rarely | 0 (0/4) | – | – | – |
| Never | 9.5 (14/147) | 20.0 (28/140) | 0.48 (0.25–0.90) | 0.48 (0.25–0.90) |
| Use of mosquito coils | | | | |
| Yes | 8.3 (3/36) | 37.5 (12/32) | 0.22 (0.06–0.79) | 0.22 (0.06–0.79) |
| No | 9.4 (11/117) | 17.3 (19/110) | 0.54 (0.26–1.14) | 0.55 (0.26–1.17) |
| Use of citronella | | | | |
| Yes | 7.1 (2/28) | 24.4 (11/45) | 0.29 (0.06–1.32) | 0.30 (0.07–1.35) |
| No | 9.6 (12/125) | 20.2 (20/99) | 0.48 (0.23–0.97) | 0.47 (0.23–0.97) |

*aPR adjusted prevalence ratio; PR, prevalence ratio; ZIKV, Zika virus; –, not applicable.
†Column n indicates the analytic sample with valid serologic results included in the sensitivity analysis. Cell denominators (No.) reflect participants with nonmissing data for each characteristic.
‡All models were adjusted for time spent at home during daylight hours, except the overall model, which was additionally adjusted for age group and sex. Adjustment approach is consistent with Sharp et al. (*9*).
§Adjusted PRs are not presented for *weekly hours at home* because this variable was used as an adjustment covariate in other models.

**Appendix Table 6.** Pooled and intervention-stratified associations between lagged mosquito abundance and Zika virus IgM seropositivity (unadjusted and adjusted), with interaction tests

| | Pooled RR (95% CI) | | | | Nonintervention RR (95% CI) | | Intervention RR (95% CI) | | p for interaction | |
|---|---|---|---|---|---|---|---|---|---|---|
| Lag, wks | Unadjusted | p value | Adjusted | p value | Unadjusted | Adjusted | Unadjusted | Adjusted | Unadjusted | Adjusted |
| 0 | 1.029 (0.996–1.063) | 0.082 | 1.028 (0.996–1.062) | 0.091 | 1.000 (0.950–1.053) | 1.000 (0.950–1.052) | 0.459 (0.174–1.211) | 0.458 (0.174–1.207) | 0.116 | 0.115 |
| 1 | 1.029 (1.004–1.055) | 0.021 | 1.028 (1.003–1.053) | 0.026 | 1.013 (0.982–1.045) | 1.012 (0.982–1.043) | 0.882 (0.328–2.371) | 0.875 (0.322–2.380) | 0.783 | 0.776 |
| 2 | 1.048 (1.015–1.083) | 0.004 | 1.044 (1.011–1.077) | 0.008 | 1.025 (0.975–1.079) | 1.019 (0.971–1.069) | 0.727 (0.344–1.535) | 0.726 (0.330–1.599) | 0.368 | 0.400 |

*Risk ratios (RRs) were estimated using Poisson regression with a log link and robust (sandwich) standard errors. Lagged mosquito abundance was modeled continuously (per +1 female per trap-week). Lag 0–2 represent mosquito abundance in the week of specimen collection (lag 0) and 1–2 weeks prior. Adjusted models include age category, sex, and hours spent at home (community) category. Stratum-specific RRs were derived from pooled models including a mosquito abundance * intervention status interaction term (not from separate stratified models). Interaction p-values are Wald tests for the interaction term using robust standard errors.

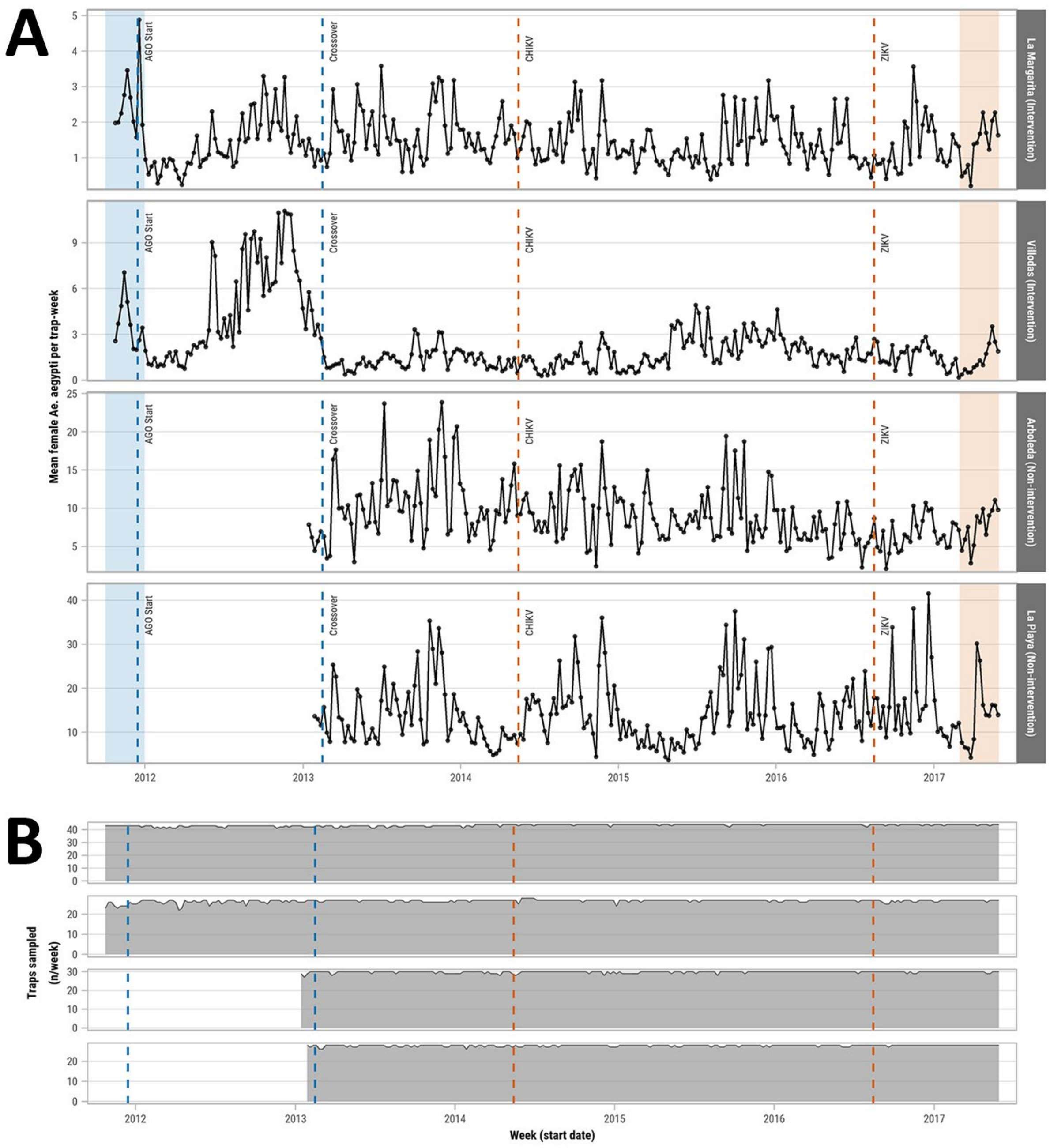


**Appendix Figure 1.** Longitudinal mosquito surveillance across study communities with pre-intervention baseline, phased AGO implementation, and arbovirus context. Weekly *Aedes aegypti* female abundance and surveillance intensity are shown for each study community (one row per community). A) Weekly mean number of female *Ae. aegypti* captured per trap-week (averaged across all traps sampled in that community-week). B) Number of traps contributing data each week (traps sampled/week) to document changes in surveillance intensity over time. Shaded bands indicate (i) the pre-intervention baseline period (October–December 2011; blue shading), used to compare communities before trap deployment, and (ii)

the human serosurvey period (March–May 2017; orange shading). During the pre-intervention baseline (Oct–Dec 2011), weekly female *Ae. aegypti* abundance was comparable between the original paired communities (La Margarita and Villodas), supporting the absence of pre-existing differences before AGO deployment. Because Arboleda and La Playa entered surveillance later as comparison communities, pre-intervention baseline comparability can be directly assessed only for the original paired communities (La Margarita and Villodas); the full longitudinal series and trap-count panel provide context for subsequent comparisons after the evaluation expanded. Routine surveillance began in late 2011 in these paired communities and continued thereafter, with additional communities entering later as the evaluation expanded. Vertical dashed lines summarize major study-design and contextual milestones reported in prior AGO evaluations. Blue dashed lines mark intervention/design changes: "AGO Start" (December 2011) denotes initiation of integrated vector control activities and AGO deployment in La Margarita during the initial controlled evaluation; "Crossover" (February 2013) denotes expansion of AGO deployment to Villodas and the transition to a sustained, area-wide evaluation framework in which Arboleda and La Playa served as comparison communities. During the initial evaluation, control trap density in La Margarita was temporarily increased from 3 to 4 traps per household (March–October 2012) after lizards were observed on sticky surfaces, and operational servicing of traps (e.g., replenishment of infusion/water and replacement of sticky surfaces) occurred throughout. In the sustained evaluation, a plastic screen was added to intervention traps to reduce domestic lizard entry, and traps were serviced on a regular schedule (approximately every 2 months), with routine sentinel monitoring maintained across sites. Orange dashed lines provide arbovirus transmission context in Puerto Rico: CHIKV denotes the onset of chikungunya emergence (May 2014), and ZIKV denotes the approximate period of peak Zika transmission (August 2016), preceding the March–May 2017 serosurvey window. Additional methodological details on phased implementation, trap density, servicing, and surveillance procedures are described in Barrera *et al*. (*1*,*2*,*5*).

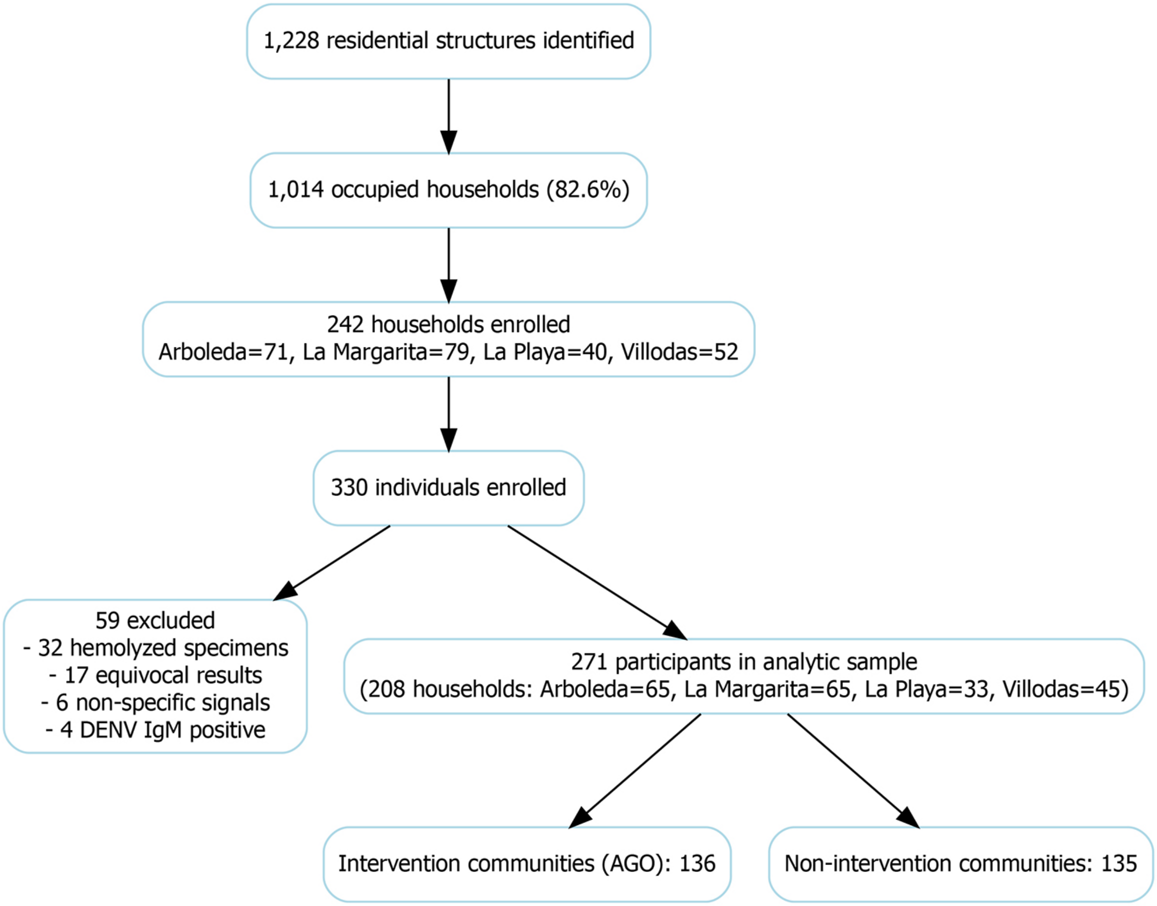


**Appendix Figure 2.** Participant flow for the Zika virus serosurvey in Salinas and Guayama, Puerto Rico, March–May 2017.

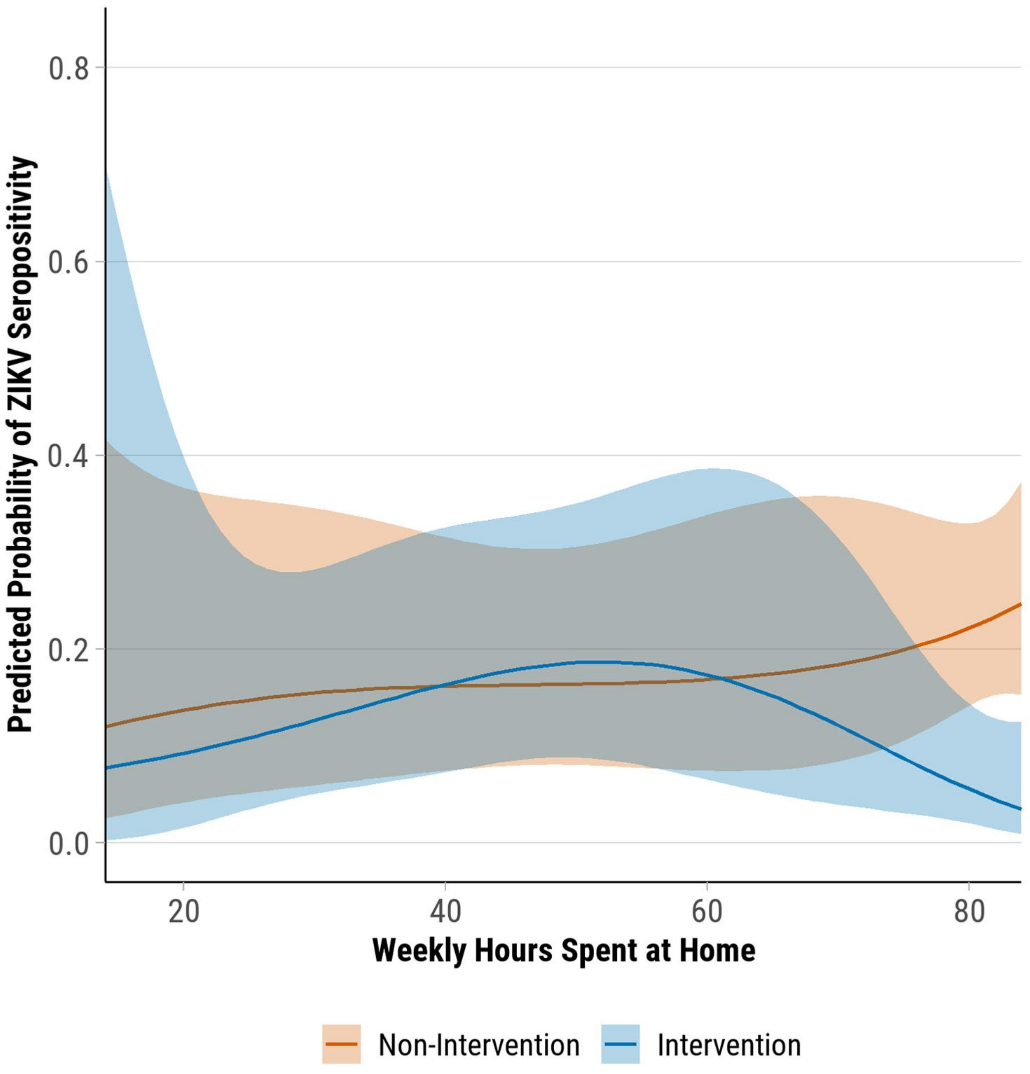


**Appendix Figure 3.** Predicted probability of ZIKV seropositivity by weekly hours spent at home, stratified by community intervention status, Puerto Rico, March–May 2017. Predicted probabilities were estimated using spline regression models with a binomial link. In nonintervention communities, the likelihood of ZIKV infection increased steadily with more time spent at home, peaking around 84 hours per week. In contrast, in intervention communities with AGOs, infection probability peaked near 52 hours per week before declining, suggesting a protective effect of the traps among participants with higher at-home exposure. Curves truncated at ≥14 hours to reflect the minimum observed value in intervention communities.
